# CD147 Mediates 5-Fluorouracil Resistance in Colorectal Cancer by Reprogramming Glycolipid Metabolism

**DOI:** 10.3389/fonc.2022.813852

**Published:** 2022-07-11

**Authors:** Shuohui Dong, Songhan Li, Xiaoyan Wang, Shuo Liang, Wenjie Zhang, Linchuan Li, Qian Xu, Bowen Shi, Zhiqiang Cheng, Xiang Zhang, Mingwei Zhong, Guangyong Zhang, Sanyuan Hu

**Affiliations:** ^1^ Department of General Surgery, Shandong Qianfoshan Hospital, Cheeloo College of Medicine, Shandong University, Jinan, China; ^2^ Department of Neonatology, Weifang Yidu Central Hospital, Weifang, China; ^3^ Department of Otolaryngology-Head and Neck Surgery, Shandong Provincial Ear-nose-throat (ENT) Hospital, Cheeloo College of Medicine, Shandong University, Jinan, China; ^4^ Department of General Surgery, The First Affiliated Hospital of Shandong First Medical University, Jinan, China; ^5^ Department of General Surgery, Qilu Hospital, Cheeloo College of Medicine, Shandong University, Jinan, China

**Keywords:** colorectal cancer, 5-fluorouracil, chemoresistance, CD147, glycolipid metabolism reprogramming

## Abstract

Chemoresistance against 5-fluorouracil (5-FU) is a major issue for colorectal cancer (CRC) patients. Increasing evidence for the roles of CD147 in glycolipid metabolic reprogramming and chemoresistance of tumor cells has emerged in recent years. However, whether CD147 contributes to 5-FU resistance in CRC and the role of abnormal glycolipid metabolism in this process remain poorly understood. We analyzed CD147 expression in primary tumor samples of CRC patients and found that upregulated CD147 correlated with decreased 5-FU chemosensitivity and an unfavorable prognosis of CRC patients. Moreover, *in vivo* and *in vitro* experiments confirmed that CD147 regulates glycolipid metabolism through two separate pathways. Mechanistically, CD147 upregulates HIF-1α-mediated glycolysis by activating the PI3K/AKT/mTOR pathway and CD147 also attenuates PPARα-mediated fatty acid oxidation by activation of the MAPK pathway. Most importantly, we found that CD147 confers 5-FU resistance in CRC *via* these glycolipid metabolic signatures. Our results demonstrated that CD147 is a potential 5-FU resistance biomarker for CRC patients and a candidate therapeutic target to restore 5-FU sensitivity of 5-FU-resistant CRC by remodeling glycolipid metabolism.

## Introduction

Colorectal cancer (CRC) causes considerable morbidity and mortality worldwide (1). Chemotherapy treatments have improved the outcomes of CRC patients, but chemoresistance remains the major cause of therapy failure (2). 5-Fluorouracil (5-FU) is one of the most efficacious and widely used chemotherapeutic agents for CRC patients (3, 4), but 5-FU resistance has become a challenge in CRC treatment (3). Therefore, identifying resistance biomarkers and effective therapeutic targets to monitor and reverse 5-FU resistance is critical.

Abnormal glycolipid metabolism plays important roles in the tumorigenesis and development of CRC (5–8). Glycolipid metabolic reprogramming provides energy to maintain the survival and proliferation of tumor cells (5, 9). It also supplies substrates for biosynthesis, which promotes malignant phenotypes that include chemoresistance (8, 10–12). Several studies have reported associations between the increased expression and activity of glycolysis-related enzymes, such as glucose transporter 1 (GLUT1), hexokinase 2 (HK2), pyruvate kinase M2 (PKM2), lactate dehydrogenase A (LDHA), and monocarboxylate transporters (MCTs), and the occurrence and development of chemoresistance (12–15). High glycolytic flux may support tumor cell resistance to chemotherapy by remodeling the energy metabolic architecture, increasing building blocks, and regulating signaling pathways (16). However, the mechanisms that underlie the increased glycolysis in chemoresistant cells remain unknown.

Lipid metabolic reprogramming is a hallmark of tumors and facilitates tumor cell escape from the cytotoxicity of chemotherapy (17, 18). Fatty acid oxidation (FAO) as a lipolytic phenotype provides considerable energy to tumor cells and is involved in regulating tumor behavior (7, 19). Upregulation of carnitine palmitoyltransferase 1 (CPT1) and other FAO-related enzymes is linked to chemoresistance in several cancers (20–22). However, other studies reported that enhanced aggressiveness is accompanied by weakening of FAO in numerous types of cancer (23). Thus, the roles of FAO in regulating the malignant features and chemoresistance of tumors are currently controversial.

CD147 is a transmembrane glycoprotein with multiple functions in diverse physiological and pathophysiological processes (24). Overexpression of CD147 is strongly linked to various malignant tumors (25). As an extracellular matrix-metalloproteinase inducer, CD147 is involved in tumor invasion and distant metastasis (26). CD147 also modulates angiogenesis by inducing tumor cells to secrete vascular endothelial growth factor (27). CD147 also plays a role in the regulation of tumor metabolism (28, 29). CD147 directs MCTs to the plasma membrane and assists their functions in transmembrane lactate transport (29). Thus, CD147 is considered a regulatory molecule of glycolysis. Additionally, a regulatory effect of CD147 on FAO and *de novo* lipid synthesis has been reported (28). CD147 is overexpressed in chemoresistant tumors of ovarian cancer (30), renal cancer (31), and Kaposi’s sarcoma (32), suggesting that CD147 is closely associated with chemoresistance.

As CD147 plays a crucial role in glycolipid metabolic reprogramming and chemoresistance, and abnormal glycolipid metabolism leads to chemotherapy failure (13), we examined whether CD147 affects 5-FU resistance in CRC by reprogramming glycolipid metabolism.

## Materials and Methods

### Antibodies and Reagents

The antibodies used in this study are shown in **Supplementary Table 1**. Detailed information on small molecule compounds and all reagents and kits is listed in **Supplementary Tables 2, 3**, respectively.

### Cell Culture and Cell Models

HCT15 and LoVo cells with STR profiling were obtained from KeyGEN BioTECH (Jiangsu, China). Cells were cultured in Roswell Park Memorial Institute-1640 (RPMI1640) medium supplemented with 10% fetal bovine serum at 37°C in 5% CO_2_. The generation of the stable acquired 5-FU resistance cell model (5FU-R cells) is described in the **Supplementary Materials**. HCT15 and LoVo cells were subjected to treatment with increasing concentrations of 5-FU, from 1×10^-8^ M to 1×10^-4^ M, for approximately 8 months. Cell survival was assessed by performing CCK-8 assays, and 5-FU resistance was identified by calculating the IC_50_ of 5-FU. To establish stable knockdown and overexpression of cell models, cells were subjected to infection with *CD147*, *HIF1A*, and *PPARA* knockdown lentiviruses, *HIF1A* overexpression lentiviruses, or their control lentiviruses. For transient *CD147* and *HIF1A* knockdown, siRNA was transfected into cells using the Lipofectamine RNA iMAX reagent. For transient *HIF1A* overexpression, overexpression plasmids were transfected into cells using Lipofectamine 3000. The sequence information is provided in the **Supplementary Table 4**.

### Mice

Athymic BALB/c nude mice (male, 4 weeks old) and NOD/SCID mice (male, 4 weeks old) were purchased from SPF Biotechnology (Beijing, China). Mice were housed in the Animal Center of Qianfoshan Hospital Affiliated to Shandong University. All procedures were approved by the Institutional Animal Care and Use Committee of Qianfoshan Hospital Affiliated to Shandong University. All animal studies complied with the relevant ethical regulations for animal testing and research.

### Patient Information and Tumor Samples

Tumor samples from CRC patients were collected from the Department of Gastrointestinal Surgery, Qianfoshan Hospital Affiliated to Shandong University, and the Department of Colorectal Surgery, Qilu Hospital of Shandong University. According to RECIST, we evaluated the patients’ response to the neoadjuvant chemotherapy was classified into four groups, including complete response (CR), partial response (PR), stable disease (SD) and progressive disease (PD), based on change in lesion size derived from imaging (contrast-enhanced CT and MRI scanning) or clinical examination. CR and PR were defined as good response, and SD and PD were defined as poor response. Clinicopathological data of patients is provided in the **Supplementary Table 5**. The Ethics Committee of Qianfoshan Hospital Affiliated to Shandong University granted approval for this study.

### CCK8 Cytotoxicity Assay

For conducting 5-FU cytotoxicity assays, 5 × 10^3^ cells, with pre-treated indicated agents, were added to 96-well plates. After adherence of the cells to the wall, they were subjected to treatment with the 5-FU for 72 h. Thereafter, they were incubated with 10% v/v of water-soluble tetrazolium salts-8 (WST-8) for 0.5-2 h, and the absorbance values of the formazan at 450 nm were measured using a microplate reader (BioRad, USA) after subjection to gentle mixing on an orbital shaker for 1 min. The percentage of viability (%) was calculated as [(absorbance of sample - absorbance of blank)/(absorbance of control - absorbance of blank) × 100%].

### Cell Sorting *via* Flow Cytometry

1×10^7^ 5FU-R CRC cells were incubated on ice with an anti-CD147 (1:200, Abcam) primary antibody for 1 h. Cells were subjected to washing steps and centrifugation, followed by incubation with Alexa-conjugated-488 secondary antibody (1:500, Proteintech) for 30 min on ice. Cells were collected after subjection to washing steps with PBS thrice, and were sorted immediately using the BD FACSAria III instrument (BD Biosciences, USA).

### RNA Extraction, Reverse Transcription PCR, and Real-Time Quantitative PCR

Total RNA extraction from cells and samples was performed using the TRIzol reagent (TaKaRa, Japan), and the concentration of RNA was measured using the NanoDrop spectrophotometer (NanoDrop Technologies, USA). RNA samples were reverse-transcribed into cDNA using the ReverTra Ace qPCR RT kit (TOYOBO, Japan). For conducting RT-qPCR, 1 μL cDNA was mixed with 0.8 μL gene-specific primers and 5 μL SYBR Green Realtime PCR Master Mix (TOYOBO, Japan), and reactions were detected by using the LightCycler 480 II instrument (Roche, Switzerland). Relative mRNA quantification was performed by adopting the ΔΔCt method, and the housekeeping gene ACTB was used as an internal reference. Detailed information on primer sequences is listed in the additional files. The relative gene expression values were estimated by performing the ΔΔCt method, and *ACTB* was used as an internal reference. The detailed primer information is provided in the **Supplementary Table 6**.

### Protein Extraction and Western Blotting

Total protein extraction from cells was performed using the RIPA buffer (Kaiji, China) containing protease inhibitor and phosphatase inhibitor (Kangwei Century, China). Cell lysates were then centrifuged, and the supernatant was boiled in 5 × loading buffer (Kangwei Century, China) at 98°C for 10 min. Proteins were resolved *via* SDS-polyacrylamide gel electrophoresis (SDS-PAGE) (BioRad, USA), and proteins in gel were transferred onto 0.45-μm PVDF membranes (Millipore, Ireland). PVDF membranes were blocked with 5% skimmed milk powder (BD Biosciences, China) in TBST for 2 h, following which PVDF membranes were incubated overnight with the primary antibody working fluid at 4°C. The primary antibodies used in the experiment were: anti-CD147 (1:5000, Abcam), anti-HIF-1α (1:500, Abcam), anti-GLUT1 (1:5000, Abcam), anti-LDHA (1:1000, Proteintech), anti-HK2 (1:1000, Abcam), anti-PKM2 (1:500, CST), anti-PI3K (1:1000, Affinity), anti-phospho-PI3K (1:1000, Affinity), anti-AKT (1:500, Abcam), anti-phospho-AKT (1:1000, Abcam), anti-mTOR (1:1000, HUABIO), anti-phospho-mTOR (1:1000, Abcam), anti-PPARα (1:500, Abcam), anti-ACOX1 (1:2000, Proteintech), anti-CPT1A (1:1000, Proteintech), anti-CPT2 (1:1000, Proteintech), anti-p38 (1:1000, HUABIO), anti-phospho-p38 (1:500, HUABIO), anti-JNK (1:1000, HUABIO), anti-phospho-JNK (1:1000, CST), anti-ERK1/2 (1:1000, HUABIO), anti-phospho-ERK1/2 (1:2000, CST), anti-β-Actin (1:5000, Proteintech). Detailed antibody information is listed in **Supplementary Table 1**. After incubation for 24 h, the PVDF membranes were incubated with the secondary antibodies (HRP-conjugated Affinipure goat anti-rabbit/mouse IgG(H+L)). Proteins were finally detected by using the LI-COR Odyssey Imager (LI-COR Biosciences, USA) using ECL (Millipore, USA) and were analyzed by using Image J software (version 1.34, USA).

### Immunocytofluorescence

1 × 10^5^ cells were cultured on 14-mm cell climbing slices. At room temperature, cells at 80% confluency were subjected to fixation in 4% paraformaldehyde (PFA) for 20 min, followed by permeabilization with 0.3% Triton X-100 for 10 min and blockade with 10% goat serum in PBS for 1 h. Anti-CD147 (1:200, Abcam) primary antibody was incubated overnight at 4°C. The following day, CoraLite594-conjugated goat anti-rabbit IgG(H+L) (1:250, Proteintech) secondary antibody was added and incubation was observed for 1.5 h at room temperature. Cell climbing slices were sealed with mounting medium containing DAPI (Abcam, USA). Image acquisition was performed using the TCS SP8 confocal laser scanning microscopy (Leica, Italy).

### Immunohistochemistry

Fresh CDX/PDX tumors and tissue samples were fixed in 4% PFA and were embedded in paraffin to prepare formalin-fixed paraffin-embedded blocks, following which the blocks were cut into 5-μm paraffin sections. The paraffin sections were sequentially subjected to dewaxing (dewaxing agent), dehydration (gradient alcohol), microwave thermal repair (sodium citrate buffer solution), and blocking (10% goat serum in PBS). The following primary antibodies were used and incubation was performed overnight at 4°C: anti-CD147 (1:500, Abcam), anti-HIF-1α (1:200, Abcam), anti-PPARα (1:100, Abcam), anti-GLUT1 (1:500, Abcam), anti-HK2 (1:100, Abcam), anti-PKM2 (1:200, CST), anti-LDHA (1:200, Proteintech), anti-ACOX1 (1:200, Proteintech), anti-CPT1A (1:100, Proteintech), anti-CPT2 (1:200, Proteintech), and anti-Ki67 (Proteintech, 1:5000). The next day, the sections were incubated with HRP-conjugated anti-rabbit/mouse IgG (ZSGB-BIO, China) and reactions were detected by performing DAB staining (ZSGB-BIO, China). The nuclei were subjected to staining procedures with hematoxylin and were differentiated in 1% acid alcohol, following which the sections were rinsed with running water. Finally, the slides were sealed with neutral gel. Images were acquired by using the Axio Scope A1 microscope (Zeiss, Germany).

### Mito-Tracker Fluorescence Staining of Mitochondria

1 × 10^5^ cells were cultured on 14-mm cell climbing slices. Cells at 80% confluency were incubated with a working solution of Mito-Tracker (Abcam, USA) for 1 h at 37°C. Cell climbing slices were then fixed, permeabilized, and sealed with mounting medium containing DAPI (Abcam, USA). Image acquisition was performed using the TCS SP8 confocal laser scanning microscopy (Leica, Italy).

### Transmission Electron Microscopy for Mitochondrial Visualization

Cells were harvested and subjected to centrifugation, following which the electron microscope fixation liquid (Servicebio, China) was added to the tube and incubation was performed for 2 h at 4°C. The fixed samples were subjected to agarose electrophoresis, followed by subjection to pre-embedding, post-fixation steps, dehydration, resin penetration and embedding, polymerization, ultrathin section preparation, and staining. Finally, images were observed and acquired using the Hitachi HT7800 transmission electron microscope (Hitachi, Japan).

### 2-NBDG Uptake Assay

We detected glucose uptake using the fluorescent glucose analog, 2-NDBG (MCE, China). When cells in 6-well plates reached 80% confluency, they were incubated with sugar-free RPMI-1640 medium (Gibco) containing 100 μM 2-NBDG for 2 h. They were then subjected to washing steps with chilled PBS and were collected in tubes. The fluorescence intensity was detected by using the BD FACSAria II (BD Biosciences, USA) flow cytometry instrument and the FITC channel. Cell debris and clusters were excluded, and the mean fluorescence intensity was calculated by using the FlowJo software (version 10.0.7, USA). As 2-NBDG exhibits green fluorescence, both *CD147* siRNA, *HIF1A* siRNA, *HIF1A*-OE plasmids, and their controls have been used in 2-NBDG detection without using any fluorescent tag.

### Lactate Release Assay

The content of lactate in supernatant was measured to quantify the extent of lactate release. The medium was replaced when cells in 24-well plates reached 80% confluency, and supernatants were collected after 24 h. The content of lactate was detected *via* colorimetric assay according to the manufacturing instructions of the kit used (KeyGEN BioTECH, China). Values of lactate were standardized according to sample protein concentrations.

### Measurement of Oxygen Consumption Rates and Extracellular Acidification Rates

1×10^4^ cells were seeded into XFe96 cell culture plates (Agilent Technologies, USA) and were cultured as per methods described earlier, followed by overnight incubation to enable cell adhesion. The next day, XFe96 cell culture plates were washed using the assay medium and were incubated at 37°C for 1 h in a CO_2_-free incubator to equilibrate the detection system. OCR and ECAR were detected using the Agilent Seahorse XFe96 extracellular flux analyzer (Agilent Technologies, USA). Seahorse XF cell mito stress test kit and Seahorse XF glycolysis stress test kit (Agilent Technologies, USA) were performed to detect the OCR and ECAR, respectively.

### Quantification of Triglyceride and Cholesterol Contents

The cells were collected and resuspended in 50% isopropanol/50% n-heptane solution. Cells were subjected to lysis using an ultrasonic cell-crushing device, followed by subjection to centrifugation to obtain the supernatant. The triglyceride assay kit (Solarbio, China) and the total cholesterol assay kit (Solarbio, China) were used to detect the content of triglyceride and cholesterol, respectively. Values of triglyceride or cholesterol were standardized according to sample protein concentrations.

### Oil Red O Staining

Cells were subjected to growth conditions for 24 h in oleic acid-containing medium. One group of cells was immediately detected *via* Oil red O staining, while another group was detected following an additional 72 h of growth in serum- and oleic acid-free medium. When suitable, a fixative solution was added into the plates for 20 min. Cells were then subjected to treatment with 60% isopropanol for 5 min. Next, neutral lipids were labeled by performing fresh Oil Red O staining and nuclei were counterstained using the Mayer Hematoxylin solution. Images were acquired by using the fluorescence microscopy Olympus IX73 (Olympus, Tokyo, Japan).

### Detection of Fatty Acid Oxidation *via* FAOBlue

When cells cultured in 24-well plates reached 80% confluency, FAOBlue (a coumarin derivative with blue fluorescence) (FUJIFILM, Japan) was added to the medium and incubation was performed for 1 h. The final FAOBlue concentration used was 5 μM. After completion of incubation, cells were subjected to washing steps with PBS and photographs were immediately acquired by using the fluorescence microscopy Olympus IX73 (Olympus, Tokyo, Japan).

### Subcutaneous Cell-Derived Xenograft Nude Mouse Model

Male athymic BALB/c nude mice were housed under SPF conditions with 12-h light/12-h dark cycles. We injected 5×10^6^ WT HCT15, shNC 5FU-R HCT15, sh*CD147* 5FU-R HCT15, *HIF1A*-OE sh*CD147* 5FU-R HCT15, and *PPARA*-KD sh*CD147* 5FU-R HCT15 into the right forelimb underarm of 4-week-old nude mice, respectively. The subcutaneous xenograft tumors were visualized one week after injection. Each group was further divided into two subgroups, and then the mice were intraperitoneally injected with 25 mg/kg 5-FU three times a week, or saline used as a control. Tumor volumes (*V*=*L* × *W* ^2^/2, where *L* represents the length and *W* denotes the width) and body weights were continuously monitored, and mice were sacrificed under anesthetization conditions established *via* inhalation of isoflurane when the tumor reached a diameter of approximately 1.5 cm.

### Subcutaneous Patient Derived Xenograft Nude Mouse Model

We obtained fresh tumor tissues from a male patient with TNM stage III rectal cancer who did not exhibit responses to 5-FU-based preoperative neoadjuvant chemotherapy. The tumor tissues were cut into pieces (approximately 3 mm) and were immediately preserved in RPMI-1640 medium containing 50% FBS and penicillin/streptomycin. We implanted the tumor pieces into the right forelimb underarm of 4-week-old NOD/scid mice, and the first generation of xenografts was harvested when the tumors reached a diameter of approximately 1.5 cm. When these xenografts were subjected to growth conditions for approximately 3 weeks, mice were divided into four groups, namely control (saline), 5-FU (administered intraperitoneally, 25 mg/kg, three times a week), AC-73 (administered intraperitoneally, 25 mg/kg, daily), and 5-FU with AC-73. Tumor volumes (*V*=*L* × *W* ^2^/2, where *L* represents the length and *W* denotes the width) and body weights were continuously monitored, and mice were sacrificed under anesthetization conditions established *via* inhalation of isoflurane when the tumor reached a diameter of approximately 1.5 cm.

### Statistical Analysis

Data are shown as means ± standard deviation (SD). Student’s t-test and one-way or two-way ANOVA were performed for comparisons. Patient survival data were evaluated by the Kaplan–Meier method with survival analysis using the Log-rank (Mantel–Cox) test. Correlation analysis was conducted using Pearson correlation analysis. *P* < 0.05 was considered statistically significant.

## Result

### Expression and Prognostic Value of CD147 in 5-FU-Resistant CRC

**Figure 1:**
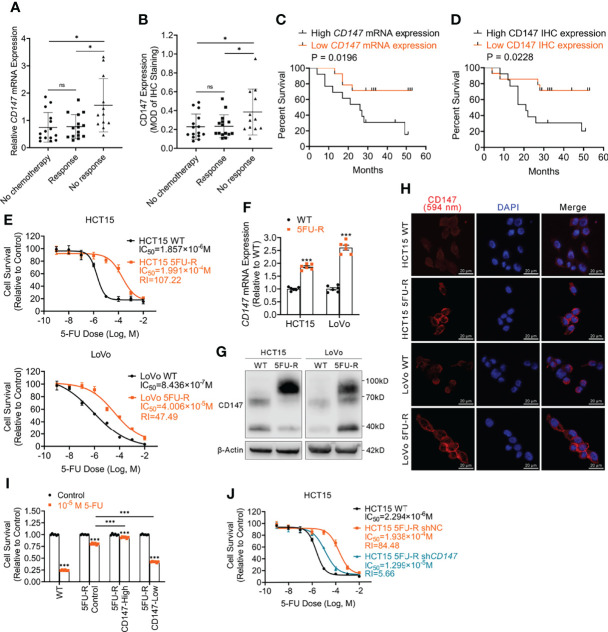
Upregulated CD147 expression correlates with 5-FU resistance and a poor prognosis of CRC patients. **(A)** Comparison of *CD147* mRNA expression in CRC patients as assessed *via* RT-qPCR. *ACTB* was used as the internal reference. **(B)** Comparison of CD147 protein expression in CRC patients as assessed *via* IHC. **(C)** Kaplan-Meier estimates of DFS of CRC patients on fluorouracil analog-based chemotherapy with high (n = 13) or low (n = 14) relative CD147 *mRNA* expression as assessed *via* RT-qPCR. **(D)** Kaplan-Meier estimates of DFS of CRC patients on fluorouracil analog-based chemotherapy with high (MOD ≥ 0.25, n = 13) or low (MOD < 0.25, n = 14) CD147 expression as assessed *via* IHC staining. **(E)** The cell survival in different concentrations of 5-FU based on the CCK-8 assay. **(F–H) (F)** RT-qPCR, **(G)** WB, and **(H)** IF analyses of the expression of CD147 in WT and 5FU-R CRC cells. **(H)** Representative images of IF staining for CD147 (red) expression and the nucleus (blue); scale bar = 20 μm. **(I)** Relative 5-FU sensitivity of 5FU-R HCT15 cells sorted *via* flow cytometry for high and low CD147 expression, compared with unsorted WT and 5FU-R HCT15 cells, as determined *via* CCK-8 assays. **(J)** Effect of *CD147* knockdown on 5-FU sensitivity of 5FU-R HCT15 cells, as assessed *via* CCK-8 assays. Data are presented as mean ± SD. Bar chart data were compared by performing the Student’s t-test or ANOVA (ns, not significant, *p < 0.05, **p < 0.01, and *** *p* < 0.001). Survival data were compared with log rank (Mantel-Cox) test results.

To explore whether CD147 expression is associated with 5-FU resistance in CRC patients, we analyzed CD147 mRNA and protein expression in primary tumor samples of three groups of TNM stage III or IV CRC patients: a no chemotherapy group (without preoperative chemotherapy), a response group (good response to preoperative fluorouracil analog–based chemotherapy), and a no response group (poor response to preoperative fluorouracil analog–based chemotherapy) (**Supplementary Table 5**). Both CD147 mRNA and protein levels were higher in the no response group compared with levels in the other groups (**Figures 1A, B**, **S1A**). Moreover, CRC patients who underwent preoperative fluorouracil analog–based chemotherapy (response and no response groups) with high CD147 mRNA or protein levels demonstrated poor disease-free survival after surgery compared with those with low CD147 expression (**Figures 1C, D**). Furthermore, we examined whether *CD147* mRNA levels showed predictive value for 5-FU chemotherapeutic efficacy using the public Gene Expression Omnibus databases (GSE69657, GSE104645). The results revealed no differences in *CD147* expression in primary tumors before chemotherapy between patients who responded and those who did not respond to fluorouracil analog–based chemotherapy (**Figure S1B**).

To investigate the role of CD147 in 5-FU-resistant CRC, we generated HCT15 5FU-R and LoVo 5FU-R CRC cells, with acquired 5-FU resistance (**Figure 1E**). Consistent with tumor sample results, CD147 mRNA and protein levels were significantly elevated in 5FU-R cells compared with those in wild-type (WT) cells (**Figures 1F–H**, **S1C**). We sorted 5FU-R cells in accordance with CD147 expression and found that 5-FU sensitivity was associated with CD147 expression (**Figures 1I**, **S1D**). Furthermore, we reduced CD147 expression levels in 5FU-R cells by shRNA knockdown (**Figure S1E**) and found that *CD147* knockdown significantly diminished 5-FU resistance in 5-FU-resistant cells (**Figures 1J**, **S1F**). These data indicate that upregulated CD147 correlates with decreased 5-FU chemosensitivity and an unfavorable prognosis of CRC patients.

### Abnormal Glycolipid Metabolism in 5FU-R CRC Cells Is Corrected by CD147 Knockdown

**Figure 2:**
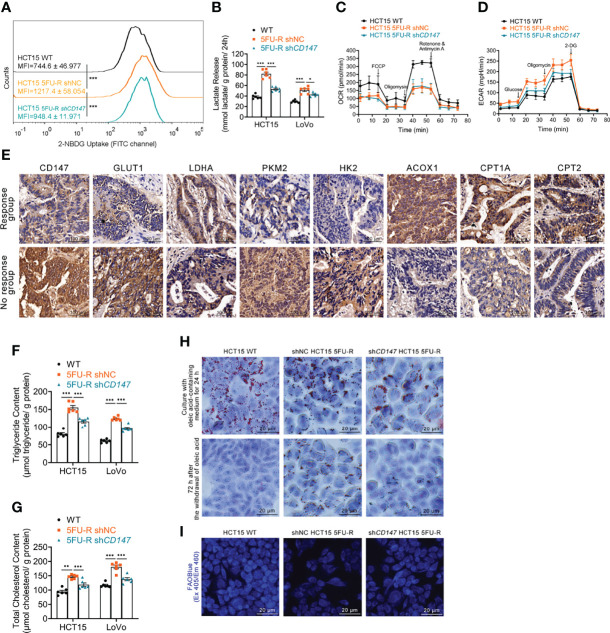
Effect of CD147 on glycolipid metabolism in 5FU-R CRC cells. **(A)** 2-NBDG signals of WT and 5FU-R HCT15 cells were quantified *via* flow cytometry. **(B)** The cell culture supernatants were assayed for lactate release using a colorimetric assay, and values were normalized by cellular protein content. **(C, D)** OCR and ECAR of WT and 5FU-R HCT15 cells were measured using Seahorse XFe96. **(C)** FCCP (1 μM), Oligomycin (1.5 μM), and rotenone/antimycin A (0.5 μM) were successively added to measure OCR, and **(D)** glucose (100 mM), oligomycin (10 μM), and 2-DG (500 mM) were successively added to measure ECAR. **(E)** Representative images of IHC staining for CD147, GLUT1, and major enzymes of glycolysis and FAO on tumor sections in CRC patients. Scale bar = 100 μm. **(F, G)** The cellular contents of **(F)** triglyceride and **(G)** total cholesterol were estimated using a colorimetric assay, and values were normalized by cellular protein content. **(H)** Oil Red O staining was performed to examine intracellular lipid droplets of HCT15 cells. Scale bar = 20 μm. **(I)** FAO was detected by using an FAOBlue probe and a fluorescence microscope. Scale bar = 20 μm. Data are presented as mean ± SD. Data were compared by performing ANOVA (ns, not significant, *p < 0.05, **p < 0.01, and ***p < 0.001).

We examined the glycolipid metabolic characteristics of CRC cells after acquisition of 5-FU resistance. The 5FU-R CRC cells consumed more glucose and produced more lactate compared with parental cells (**Figures 2A, B**, **S2A**). Higher glucose/lactate fluxes were accompanied by a decreased oxygen consumption rate and an increased ECAR (**Figures 2C, D**, **S2B, C**). We also observed reduced mitochondrial respiration and enhanced glycolysis in 5FU-R cells (**Figure S2D**). Expressions of GLUT1 and major enzymes of glycolysis were also elevated in tumor samples of the no response group compared with those in tumors of the response group (**Figure 2E**). These results suggested that 5FU-R cells had switched from oxidative phosphorylation to glycolysis.

We next assessed lipid metabolism in 5FU-R cells. Increased intracellular triglyceride and cholesterol levels (**Figures 2F, G**), decreased consumption of intracellular lipids (**Figures 2H**, **S2E**), and attenuated FAO (**Figures 2I**, S2F) were observed in 5FU-R CRC cells compared with those in WT cells. The expression of major FAO enzymes was downregulated in 5-FU-insensitive patients compared with 5-FU-sensitive patients (**Figure 2E**).

Notably, abnormal glycolipid metabolism in 5FU-R cells was partly corrected by *CD147* knockdown (**Figures 2A–D, F–I**, **S2A–F**). Attenuated 5-FU resistance of *CD147* knockdown 5FU-R CRC cells was partially be reversed by the glycolytic activators, Mitapivat (a selective PKM2 activator) or Oligomycin (an ATP synthase/complex V inhibitor, could be used to release glycolytic reserve) (**Figures S2G, H**). Furthermore, 5-FU resistance reversal after *CD147* knockdown was partially restored by FAO inhibitors, 10,12-Tricosadiynoic acid (a selective ACOX1 inhibitor) or etomoxir sodium salt (a reversible CPT-1 inhibitor), in *CD147* knockdown 5FU-R cells (**Figures S2I, J**). These data suggest that CD147 mediates glycolipid metabolic reprogramming in 5-FU-resistant CRC.

Decreased oxidative phosphorylation and FAO in 5FU-R cells indicated disruption of mitochondrial oxidative functions. We confirmed this by visualizing mitochondria with Mito-Tracker staining, which showed reduced mitochondrial contents (**Figure S2K**). Transmission electron microscopy images revealed destruction of the mitochondrial ultrastructure in 5FU-R cells (**Figure S2L**).

### CD147 Enhances Glycolysis by Upregulation of HIF-1α in 5-FU-Resistant CRC Cells

**Figure 3:**
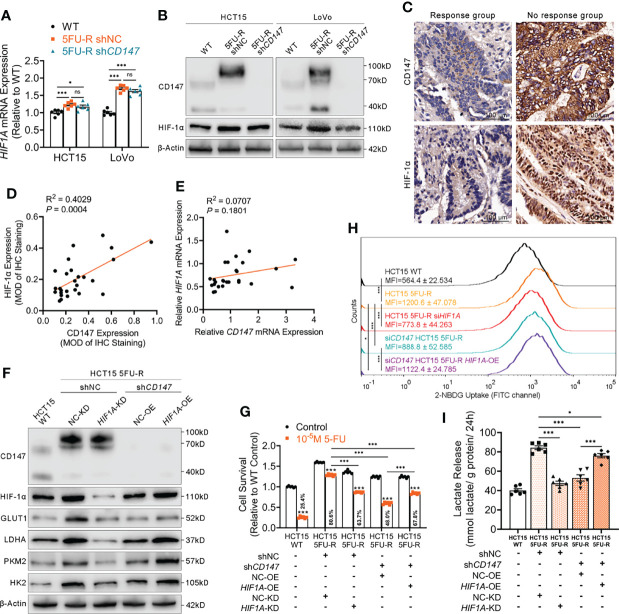
CD147 enhances glycolysis by upregulating HIF-1α expression. **(A, B) (A)** RT-qPCR and **(B)** WB analyses of the expression levels of HIF-1α in WT, 5FU-R, and sh*CD147* 5FU-R CRC cells. **(C)** Representative images of IHC staining for CD147 and HIF-1α expression on tumor sections in CRC patients. Scale bar = 100 μm. **(D)** Correlations between protein levels of CD147 and HIF-1α in 27 tumor specimens of CRC patients treated with preoperative fluorouracil analog-based chemotherapy, as estimated by performing Pearson correlation analysis. Protein levels were quantified by IHC staining. **(E)** Correlations between mRNA levels of *CD147* and *HIF1A* in 27 tumor specimens of CRC patients as previously described, as estimated by performing Pearson correlation analysis. **(F)** WB analyses of CD147, HIF-1α, GLUT1, and glycolytic enzymes in WT HCT15 cells, 5FU-R HCT15 cells subjected to treatment with sh*HIF1A* or control shRNA, and sh*CD147* HCT15 5FU-R cells subjected to treatment with *HIF1A*-OE lentivirus or control lentivirus. **(G)** Relative 5-FU sensitivity of the indicated HCT15 cells, as determined *via* CCK-8 assays. **(H)** 2-NBDG signals were quantified *via* flow cytometry in WT HCT15 cells, 5FU-R HCT15 cells subjected to treatment with si*HIF1A* or control siRNA, and si*CD147* HCT15 5FU-R cells subjected to treatment with *HIF1A*-OE plasmids or control plasmids. **(I)** The cell culture supernatants of the indicated HCT15 cells were assayed for lactate release using a colorimetric assay, and values have been normalized by cellular protein content. Data are presented as mean ± SD. Data were compared by performing ANOVA (ns, not significant, *p < 0.05, **p < 0.01, and ***p < 0.001). Correlation analysis was conducted using Pearson correlation analysis.

HIF-1α is the most important regulator of glycolysis (33). We examined HIF-1α levels in CRC cells with different CD147 expression and observed significantly higher HIF-1α mRNA and protein levels in 5FU-R cells compared with that in WT cells (**Figures 3A, B**). HIF-1α protein expression decreased after *CD147* knockdown in 5FU-R cells, with no changes in HIF-1α mRNA level (**Figures 3A, B**). We further examined the expressions of CD147 and HIF-1α in 27 tumor specimens of CRC patients treated with preoperative fluorouracil analog–based chemotherapy. CD147 and HIF-1α expressions in protein level showed a significant positive correlation (**Figures 3C, D**), while no correlation was observed with mRNA level (**Figure 3E**).

We next investigated whether CD147 enhanced glycolysis through HIF-1α in 5-FU-resistant CRC cells. *HIF1A* knockdown in 5FU-R cells resulted in decreased 5-FU resistance, downregulated GLUT1 and glycolytic enzyme levels, decreased glucose uptake, and reduced lactate release. *CD147* knockdown attenuated 5-FU resistance and weakened the phenotypes of glycolysis in 5FU-R cells, while these reductions were partially restored by *HIF1A* overexpression (**Figures 3F–I**, **S3A–D**).

### CD147 Upregulates HIF-1α Through the Activated PI3K/AKT/mTOR Signaling Pathway in 5FU-R CRC Cells

**Figure 4:**
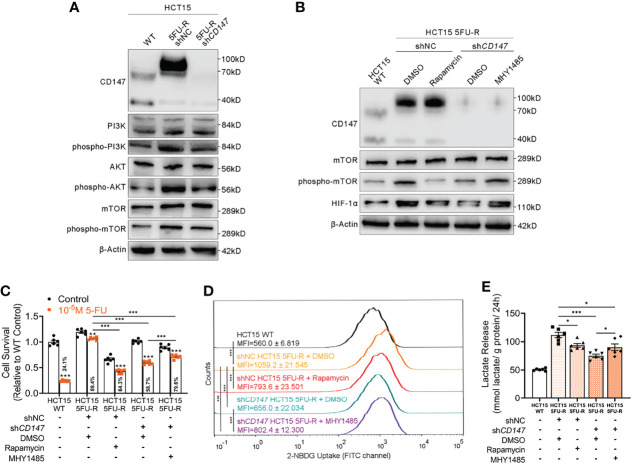
CD147 upregulates HIF-1α expression through the activated PI3K/AKT/mTOR signaling pathway. **(A)** WB analyses of the expression levels of PI3K, phospho-PI3K, AKT, phospho-AKT, mTOR, and phospho-mTOR in WT, 5FU-R, and sh*CD147* 5FU-R HCT15 cells. **(B)** WB analyses of CD147, mTOR, phospho-mTOR, and HIF-1α in WT HCT15 cells, 5FU-R HCT15 cells subjected to treatment with 50 nM rapamycin or DMSO (control), and sh*CD147* HCT15 5FU-R cells subjected to treatment with 10 μM MHY1485 or DMSO (control). **(C)** Relative 5-FU sensitivity of the indicated HCT15 cells, as determined *via* CCK-8 assays. **(D)** 2-NBDG signals of the indicated HCT15 cells were quantified *via* flow cytometry. **(E)** The cell culture supernatants of the indicated HCT15 cells were assayed for lactate release using a colorimetric assay, and values have been normalized by cellular protein content. Data are presented as mean ± SD. Data were compared by performing ANOVA (ns, not significant, *p < 0.05, **p < 0.01, and ***p < 0.001).

Several studies have implicated the PI3K/AKT/mTOR signaling pathway in glucose metabolism and chemotherapy resistance (34, 35). Additionally, the PI3K/AKT/mTOR pathway is activated by CD147 (36, 37). Therefore, we hypothesized that CD147-mediated activation of the PI3K/AKT/mTOR pathway may upregulate HIF-1α, which further enhances glycolysis and induces 5-FU resistance in 5-FU-resistant CRC. The phosphorylated levels of PI3K, AKT, and mTOR were elevated in 5FU-R cells compared with those of WT cells, whereas *CD147* knockdown in 5FU-R cells suppressed activation of the PI3K/AKT/mTOR pathway (**Figures 4A**, **S4A**). Furthermore, 5FU-R cells treated with rapamycin (38), an mTOR inhibitor, showed downregulated HIF-1α level, attenuated 5-FU resistance, and decreased glucose uptake and lactate release. Moreover, attenuated 5-FU resistance and decreased glycolytic flux in 5FU-R cells caused by *CD147* knockdown were restored by treatment with MHY1485 (39), an mTOR activator (**Figures 4B-E**, **S4B–E**).

### CD147 Suppresses FAO *via* Downregulation of PPARα in 5-FU-Resistant CRC Cells

**Figure 5:**
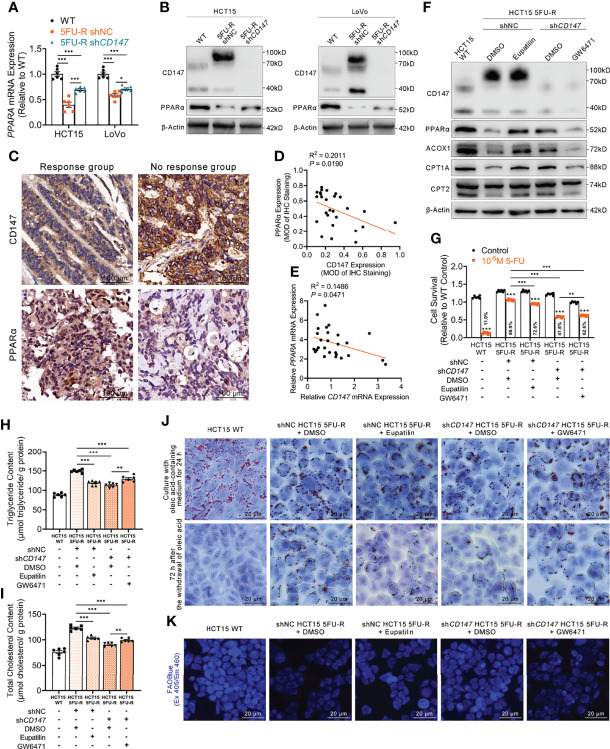
CD147 suppresses FAO by downregulating PPARα expression. **(A, B) (A)** RT-qPCR and **(B)** WB analyses of the expression levels of PPARα in WT, 5FU-R, and sh*CD147* 5FU-R CRC cells. **(C)** Representative images of IHC staining for CD147 and PPARα expression on tumor sections in CRC patients. Scale bar = 100 μm. **(D)** Correlations between protein levels of CD147 and PPARα in 27 tumor specimens of CRC patients as previously described, as determined *via* Pearson correlation analysis. Protein levels were quantified by IHC staining. **(E)** Correlations between mRNA levels of *CD147* and *PPARA* in 27 tumor specimens of CRC as previously described, as estimated *via* Pearson correlation analysis. **(F)** WB analyses of CD147, PPARα, and FAO-related enzymes in WT HCT15 cells, 5FU-R HCT15 cells subjected to treatment with 50 μM eupatilin or DMSO (control), and sh*CD147* HCT15 5FU-R cells subjected to treatment with 25 μM GW6471 or DMSO (control). **(G)** Relative 5-FU sensitivity of the indicated HCT15 cells, as determined *via* CCK-8 assays. **(H, I)** The cellular contents of **(H)** triglyceride and **(I)** total cholesterol of the indicated HCT15 cells were assayed using a colorimetric assay, and values have been normalized by cellular protein content. **(J)** Oil Red O staining was performed to examine intracellular lipid droplets of the indicated HCT15 cells. Scale bar = 20 μm. **(K)** FAO was detected by using an FAOBlue probe and a fluorescence microscope. Scale bar = 20 μm. Data are presented as mean ± SD. Data were compared by performing ANOVA (ns, not significant, *p < 0.05, **p < 0.01, and ***p < 0.001).

A markedly attenuated FAO rate and accumulated intracellular lipids were characteristic changes of lipid metabolism in 5FU-R CRC cells. The PPAR transcription factor promotes the transcription of key factors in FAO (40). A negative correlation between the expressions of CD147 and PPARα was reported in hepatocellular carcinoma (28). We thus hypothesized that CD147-mediated abnormal lipid metabolism in 5-FU-resistant CRC may be mediated through downregulated PPARα. PPARα mRNA and protein levels were significantly reduced in 5FU-R cells compared with those in WT cells, whereas PPARα levels were increased in *CD147* knockdown 5FU-R cells (**Figures 5A, B**). Additionally, PPARα expression was negatively related to CD147 at both mRNA and protein levels in 27 specimens from CRC patients treated with preoperative fluorouracil analog–based chemotherapy (**Figures 5C–E**).

Next, we examined whether the effect of CD147 on PPARα contributed to the regulation of FAO in 5FU-R cells. After treating 5FU-R cells with eupatilin (41), a PPARα agonist, we observed enhanced 5-FU sensitivity, higher FAO enzyme expressions, decreased triglyceride and cholesterol contents, increased consumption of intracellular lipids, and accelerated FAO compared with those of DMSO-treated 5FU-R cells. Conversely, 5-FU resistance reversal and acceleration of intracellular lipid metabolism after *CD147* knockdown were partially restored by GW6471 (a PPARα antagonist) (42) in *CD147* knockdown 5FU-R cells (**Figures 5F–K**, **S5A–F**).

### CD147 Downregulates PPARα by Activating the MAPK Signaling Pathway in 5FU-R CRC Cells

**Figure 6:**
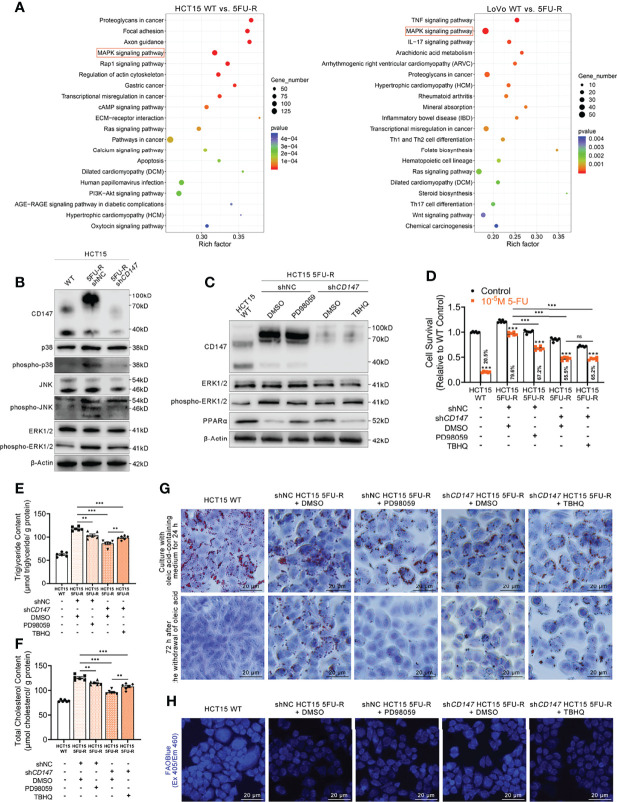
CD147 downregulates PPARα expression through the activated MAPK signaling pathway. **(A)** Enrichment analysis of KEGG pathways using transcriptome data obtained for WT and 5FU-R CRC cells. **(B)** WB analyses of the expression levels of p38, phospho-p38, JNK, phospho-JNK, ERK, and phospho-ERK in WT, 5FU-R, and sh*CD147* 5FU-R HCT15 cells. **(C)** WB analyses of CD147, ERK, phospho-ERK, and PPARα in WT HCT15 cells, 5FU-R HCT15 cells subjected to treatment with 50 μM PD98059 or DMSO (control), and sh*CD147* HCT15 5FU-R cells subjected to treatment with 20 μM TBHQ or DMSO (control). **(D)** Relative 5-FU sensitivity of the indicated HCT15 cells, as determined *via* CCK-8 assays. **(E, F)** The cellular contents of **(E)** triglyceride and **(F)** total cholesterol of the indicated HCT15 cells were assayed using a colorimetric assay, and values have been normalized by cellular protein content. **(G)** Oil Red O staining was performed to examine intracellular lipid droplets of the indicated HCT15 cells. Scale bar = 20 μm. **(H)** FAO was detected by using an FAOBlue probe and a fluorescence microscope. Scale bar = 20 μm. Data are presented as mean ± SD. Data were compared by performing ANOVA (ns, not significant, *p < 0.05, **p < 0.01, and ***p < 0.001).

Activation of the MAPK signaling pathway decreases PPAR transcriptional activity (43). Regulation of ERK, a MAPK family member, by CD147 has also been reported in cancer and atherosclerosis (44, 45). Enrichment analysis of KEGG pathways on transcriptome data of WT and 5FU-R CRC cells included the MAPK signaling pathway (**Figure 6A**). Therefore, we suspected that MAPK signaling might play a role in CD147 negative regulation of PPARα. JNK, ERK, and p38 phosphorylated levels were markedly upregulated in 5FU-R cells, whereas *CD147* knockdown in 5FU-R cells suppressed the phosphorylation of these MAPK-related molecules (**Figures 6B**, **S6A**). We next examined whether ERK mediated the negative regulation of PPARα by CD147, because it showed the most pronounced increase in phosphorylation in 5FU-R cells. In 5FU-R cells treated with PD98059 (an ERK1/2 signaling inhibitor) (46), PPARα expression was upregulated and the sensitivity to 5-FU and FAO was also enhanced. Conversely, treatment of *CD147* knockdown 5FU-R cells with TBHQ (an ERK activator) (47) reversed the *CD147* knockdown-induced resistance and lipid metabolic phenotypes (**Figures 6C–H**, **S6B–G**).

Furthermore, we detected whether a crosstalk exists between glycolysis and FAO regulations in 5FU-R CRC cells. To determine whether HIF-1α affect MAPK/PPARα axis in 5FU-R cells, WB analyses of ERK, phospho-ERK and PPARα in WT HCT15 cells, 5FU-R HCT15 cells subjected to treatment with sh*HIF1A* or control shRNA, and sh*CD147* HCT15 5FU-R cells subjected to treatment with *HIF1A*-OE lentivirus or control lentivirus. The results indicated that HIF-1α did not notably affect the MAPK/PPARα axis in 5FU-R cells (**Figure S6H**). Additionally, to detect the influence of PPARa on PI3K/AKT/mTOR/HIF-1α axis in 5FU-R cells, WB analyses of mTOR, phospho-mTOR and HIF-1α in WT HCT15 cells, 5FU-R HCT15 cells subjected to treatment with eupatilin or DMSO (control), and sh*CD147* HCT15 5FU-R cells subjected to treatment with GW6471 or DMSO (control). The results showed that there is a slightly negative regulation of PI3K/AKT/mTOR/HIF-1α axis by PPARa in 5FU-R cells (**Figure S6I**).

### Upregulated CD147 Confers 5-FU Resistance in CRC *via* Glycolipid Metabolic Reprogramming

**Figure 7:**
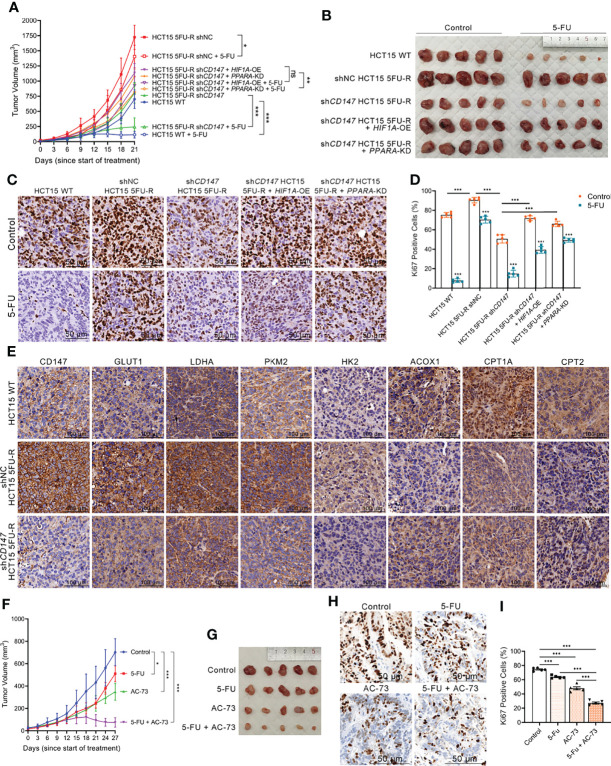
Upregulated CD147 confers 5-FU resistance in CRC *via* glycolipid metabolic reprogramming. **(A, B) (A)** Tumor growth curves and **(B)** dissected tumors of subcutaneous xenograft tumor formation with the indicated HCT15 cells, followed by treatment with 5-FU (administered intraperitoneally, 25 mg/kg, three times a week) or saline (control). **(C, D) (C)** Representative images of IHC staining for Ki-67 expression on subcutaneous xenograft tumors of the indicated HCT15 cells; scale bar = 50 μm. **(D)** Percentage of Ki-67-positive cells. **(E)** Representative images of IHC staining for determining expression of CD147, GLUT1, and major enzymes of glycolysis and FAO on subcutaneous xenograft tumors of the indicated HCT15 cells. Scale bar = 100 μm. **(F, G) (F)** Tumor growth curves and **(G)** dissected tumors of PDX NOD/scid mice, followed by treatment with 5-FU (administered intraperitoneally, 25 mg/kg, three times a week), AC-73 (administered intraperitoneally, 25 mg/kg, daily), or saline (control). **(H, I) (H)** Representative images of IHC staining for Ki-67 expression on tumor sections of PDX models; scale bar = 50 μm. **(I)** Percentage of Ki-67-positive cells. Data are presented as mean ± SD. Data were compared by performing ANOVA (ns, not significant, *p < 0.05, **p < 0.01, and ***p < 0.001).

To evaluate the effect of CD147 on 5-FU resistance of CRC cells *in vivo*, WT, shNC, and sh*CD14*7 5FU-R HCT15 cells were injected subcutaneously into nude mice. After 1 week, tumor-bearing mice were treated with 5-FU or saline. Tumors in 5FU-R cell–bearing mice grew faster and were insensitive to treatment compared with those in WT cell–bearing mice (**Figures 7A, B**). Tumors from 5FU-R cell-bearing mice exhibited higher Ki-67-positive rates (**Figures 7C, D**) and higher expression of CD147, glycolytic and FAO enzymes (**Figure 7E**). Conversely, tumors from *CD147* knockdown 5FU-R cell–bearing mice had decelerated growth, increased 5-FU sensitivity, and reversed glycolipid metabolism compared with controls (**Figures 7A–E**). We also established a patient-derived xenograft mouse model from a male patient with TNM stage III rectal cancer who did not respond to 5-FU-based preoperative neoadjuvant chemotherapy. Mice were treated with a combination of 5-FU and AC-73 (a CD147 inhibitor) (48), which significantly inhibited tumor growth, proliferation, and glycolipid metabolism compared with 5-FU alone (**Figures 7F–J**). Mouse bodyweight was unaffected by 5-FU, but the AC-73 group showed weight loss, suggesting that AC-73 treatment may have potential risks (**Figures S7A, B**).

Considering that metabolic reprogramming leads to changes in tumor behavior (10), we hypothesized that CD147-mediated 5-FU resistance in CRC may be mediated through glycolipid metabolic reprogramming. We established cell models with *HIF1A* overexpression and *PPARA* knockdown in *CD147* knockdown 5FU-R HCT15 cells. Notably, the inhibitory effects of *CD147* knockdown were partially reversed by *HIF1A* overexpression or *PPARA* knockdown (**Figures 7A–E**). These results showed that CD147 confers 5-FU resistance in CRC by glycolipid metabolic reprogramming.

## Discussion

CRC resistance to 5-FU is challenging in the clinic (3). In the 5FU-R CRC cell models and patient samples, CD147 enhanced HIF-1α-induced glycolysis by activating the PI3K/AKT/mTOR signaling pathway and suppressed PPARα-mediated FAO by activating the MAPK signaling pathway. Our findings showed that overexpressed CD147 confers 5-FU resistance to CRC by reprogramming glycolipid metabolism. We demonstrated that CD147 is a potential 5-FU resistance biomarker for CRC patients and a valuable therapeutic target for 5-FU-resistant CRC.

Several enzymes and regulators of glycolysis are associated with chemoresistance (49–51). Our findings showed that CD147 promoted upregulation of glycolytic enzymes, higher glucose uptake, lactate release, and ECAR in 5-FU-resistant CRC cells. Glucose addiction and enhanced aerobic glycolysis, the so-called “Warburg effect”, are metabolic features of tumor cells (52). We found that 5-FU-resistant CRC cells shifted from oxidative phosphorylation to glycolysis, which was consistent with the observed disruption of mitochondria. Enhanced glycolysis may therefore be a compensatory change for the insufficient energy production from mitochondrial dysfunction. Such adaptive changes in 5-FU-resistant CRC cells are important for energy supply but also generate intermediates of macromolecule biosynthesis. HIF-1α is a major regulator of glycolysis and overexpressed HIF-1α is associated with chemoresistance in pancreatic cancer (13). We identified the contribution of HIF-1α-mediated glycolysis to 5-FU resistance in CRC and confirmed that HIF-1α was regulated by CD147. HIF-1α is regulated by oxygen-dependent prolyl hydroxylases (53). However, we found that CD147 regulated HIF-1α protein levels through phosphorylation of the PI3K/AKT/mTOR pathway, which represents a non-classical and oxygen-independent regulation mode.

Many studies of tumor metabolism have concentrated on the Warburg effect, glutaminolysis, and *de novo* fatty acid synthesis (54, 55). Studies on FAO have been limited, with conflicting results. Some research revealed that FAO is an essential ATP source in ovarian cancer and leukemia to maintain survival of tumor cells (56, 57). FAO enzymes may be upregulated by oncoproteins such as c-Myc (58), suggesting that activated FAO is a part of oncogene-associated signaling pathways. However, other studies demonstrated that weakened FAO correlated with increased tumor malignancy (23, 28). A possible explanation is that accumulated intracellular lipids are not directly involved in the energy supply and instead shunt into pathways for biomolecule synthesis. In our cell models and patient samples, FAO was significantly slower with the development of 5-FU resistance in CRC. Our study raises the possibility that reduced FAO results from an impaired mitochondrial oxidative capacity in 5-FU-resistant CRC. However, as other metabolic pathways may provide adequate energy, decreased FAO would not affect the survival of 5-FU-resistant cells. Conversely, excess lipids may be involved in other essential functions and play a critical role in 5-FU resistance. PPARα is a critical regulator of enzymes in FAO (40) and our results are in accordance with studies indicating that PPARα has a beneficial effect against cancer (59). Interestingly, we found that PPARα-mediated FAO was negatively regulated by CD147 in 5-FU-resistant CRC. Several reports showed that CD147 exerts a prominent regulatory effect on the MAPK pathway (44, 45). Our findings demonstrate that CD147 has a negative regulatory function in PPARα-mediated FAO by activation of the MAPK signaling pathway.

The most clinically relevant finding is the potential diagnostic and therapeutic value of CD147 for 5-FU-resistant CRC. We found that CD147 may be an efficient biomarker for assessment and prognosis of CRC patients treated with fluorouracil analog–based chemotherapy. CD147 is involved in multiple tumor processes, including invasion, metastasis, angiogenesis, and epithelial–mesenchymal transition, which are associated with the formation of circulating tumor cells (CTCs) (60, 61). Further study is needed to assess the association between CD147-positive CTCs and 5-FU resistance in CRC and explore a “liquid biopsy” approach for real-time monitoring of chemotherapy response. Additionally, we demonstrated that targeting CD147 may be a promising strategy to increase 5-FU sensitivity in 5-FU-resistant CRC *in vitro* and *in vivo.* We found that AC-73, a small molecule CD147 inhibitor (48), reversed 5-FU resistance. However, body weights were significantly lower after AC-73 treatment, raising concerns of safety. Therefore, CD147-targeted small molecule inhibitors that are suitable and safe for *in vivo* use are needed.

An anti-CD147 monoclonal antibody (MEM-M6/1) induces cell death in colon cancer by blocking CD147 and MCT1 binding (62). Considering the high flux of lactate transmembrane transport in 5-FU-resistant CRC cells, MEM-M6/1 may be a good alternative for 5-FU-resistant CRC patients. Licartin, a ^131^I-radioisotope-labeled anti-CD147 monoclonal antibody (metuximab), was approved for hepatocellular carcinoma treatment by the Chinese State Food and Drug Administration (63) and increased the chemosensitivity of pancreatic cancer to gefitinib and gemcitabine (64). We will evaluate Licartin for its possible use in 5-FU-resistant CRC treatment. Furthermore, we observed 5FU-R CRC cells had a higher expression of HG-CD147 (highly glycosylated CD147) compared to WT CRC cells. In present study, our main focus was on the influence of total CD147 on 5-FU resistance and glycolipid metabolism. The functional research of CD147 is also affected by glycosylation modification, and we will carry out related work in this field to identify the influence of glycosylated CD147 on chemoresistance of CRC.

CD147 is involved in cell proliferation, migration, invasion, and angiogenesis (25–27). Our findings suggested that CD147 also mediates 5-FU resistance in CRC through increased glycolysis and decreased FAO. We identified CD147 as a potential novel biomarker for accurate assessment of 5-FU resistance and evaluation of prognosis in CRC patients treated with 5-FU. Our results revealed that suppressing the expression of CD147 may be a therapeutic strategy to restore 5-FU sensitivity of 5-FU-resistant CRC by remodeling glycolipid metabolism. However, we are aware of chemoresistance is a complicated event that cannot be explained by single molecules or simple signaling pathways. In present study, we investigated CD147 as a core molecule and explored the upstream and downstream regulatory mechanisms in 5-FU-resistant CRC, but it is clearly not sufficient for comprehensively reveal the pathophysiological state of chemoresistance. More efforts will be made into sets in future which make up a complete study system of 5-FU-resistant CRC.

## Data Availability Statement

The original contributions presented in the study are included in the article/**Supplementary Material**. further inquiries can be directed to the corresponding author.

## Ethics Statement

The studies involving human participants were reviewed and approved by the Ethics Committee of Qianfoshan Hospital Affiliated to Shandong University. The patients/participants provided their written informed consent to participate in this study. The animal study was reviewed and approved by the Institutional Animal Care and Use Committee of Qianfoshan Hospital Affiliated to Shandong University. Written informed consent was obtained from the individual(s) for the publication of any potentially identifiable images or data included in this article.

## Author Contributions

Conceptualization, SH, SD, GZ. Methodology, SD, SOL, XW, MZ and WZ. Validation, SOL, LL and QX. Data analysis, BS, SD. Writing original draft preparation, ZC and XZ. All authors have read and agreed to the published version of the manuscript.

## Funding

This work was supported by National Natural Science Foundation of China (82070869, 81770795, 81900705) and National Key Research and Development Program of China (2016YFC0106003).

## Conflict of Interest

The authors declare that the research was conducted in the absence of any commercial or financial relationships that could be construed as a potential conflict of interest.

## Publisher’s Note

All claims expressed in this article are solely those of the authors and do not necessarily represent those of their affiliated organizations, or those of the publisher, the editors and the reviewers. Any product that may be evaluated in this article, or claim that may be made by its manufacturer, is not guaranteed or endorsed by the publisher.
