# Supplementary material for: CD147 Mediates 5-Fluorouracil Resistance in Colorectal Cancer by Reprogramming Glycolipid Metabolism
Source: Front Oncol. 2022 Jul 11;12:813852. doi: 10.3389/fonc.2022.813852 (PMC9309564; doi:10.3389/fonc.2022.813852)
Supplement: Supplementary file 1 [file DataSheet_1.doc]

**Supplementary Figures**

**Title:** CD147 mediates 5-fluorouracil resistance in colorectal cancer by reprogramming glycolipid metabolism

**Authors’ names:** ShuoHui Dong^1^, SongHan Li^1^, XiaoYan Wang^2^, Shuo Liang^3^, WenJie Zhang^1^, LinChuan Li^4^, Qian Xv^1^, BoWen Shi^1^, ZhiQiang Cheng^5^, Xiang Zhang^5^, MingWei Zhong^4^, GuangYong Zhang^4^, SanYuan Hu^1*^

**Authors’ affiliations:**

^1^Department of General Surgery, Shandong Qianfoshan Hospital, Cheeloo College of Medicine, Shandong University, Jinan, China.

^2^Department of Neonatology, Weifang Yidu Central Hospital, Weifang, China.

^3^Department of Otolaryngology-Head and Neck Surgery, Shandong Provincial ENT Hospital, Cheeloo College of Medicine, Shandong University, Jinan, China.

^4^Department of General Surgery, The First Affiliated Hospital of Shandong First Medical University, Jinan, China

^5^Department of General Surgery, Qilu Hospital, Cheeloo College of Medicine, Shandong University, Jinan, China.

**Corresponding author:** SanYuan Hu, Department of General Surgery, Shandong Qianfoshan Hospital, Cheeloo College of Medicine, Shandong University, Jinan, Shandong 250014, China. Email: drsanyuanhu@163.com. Tel.: +86 18663738139.

**
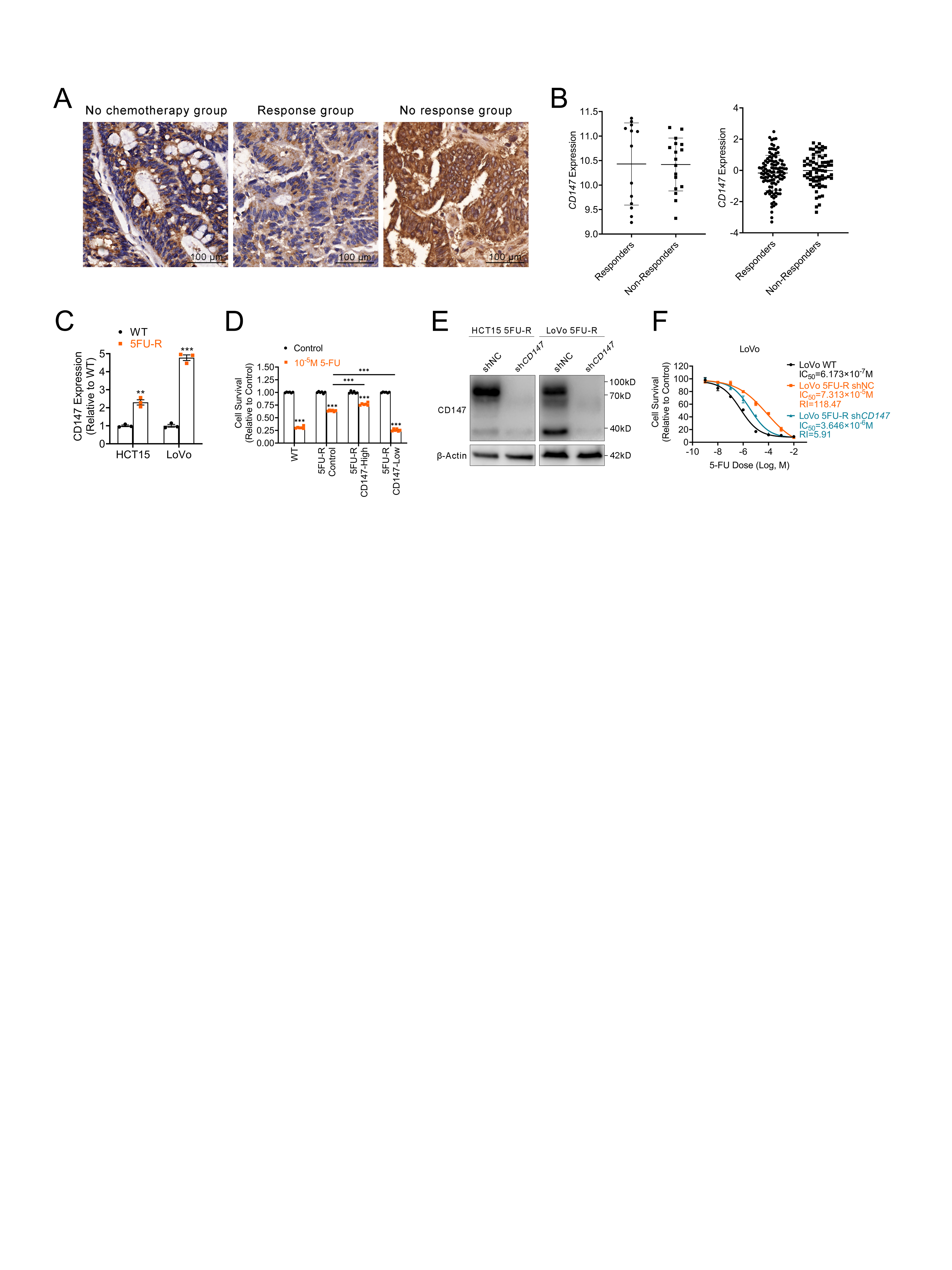
**

**Supplementary Figure 1 (related to Figure 1)**

1. Representative images of IHC staining for CD147 expression on tumor sections in CRC patients. Scale bar = 100 μm.
2. *CD147* mRNA expressions were obtained and compared from 2 GEO datasets (GSE69657, GSE104645).
3. Statistical plots of WB analyses (CD147) in WT and 5FU-R CRC cells.
4. Relative 5-FU sensitivity of 5FU-R LoVo cells sorted via flow cytometry for high and low CD147 expression, compared with unsorted WT and 5FU-R LoVo cells, as determined via CCK-8 assays.
5. Knockdown of *CD147* by shRNA validated via western blotting.
6. Effect of *CD147* knockdown on 5-FU sensitivity of 5FU-R LoVo cells. Viability was measured by performing the CCK-8 assay after subjecting cells to treatment with increasing concentrations of 5-FU for 72 h.

Data are presented as mean ± SD. Bar chart data were compared by performing the Student’s *t*-test or ANOVA (ns = not significant, * *p* < 0.05, ** *p* < 0.01, and *** *p* < 0.001). Source data are provided as a Source Data file.

**
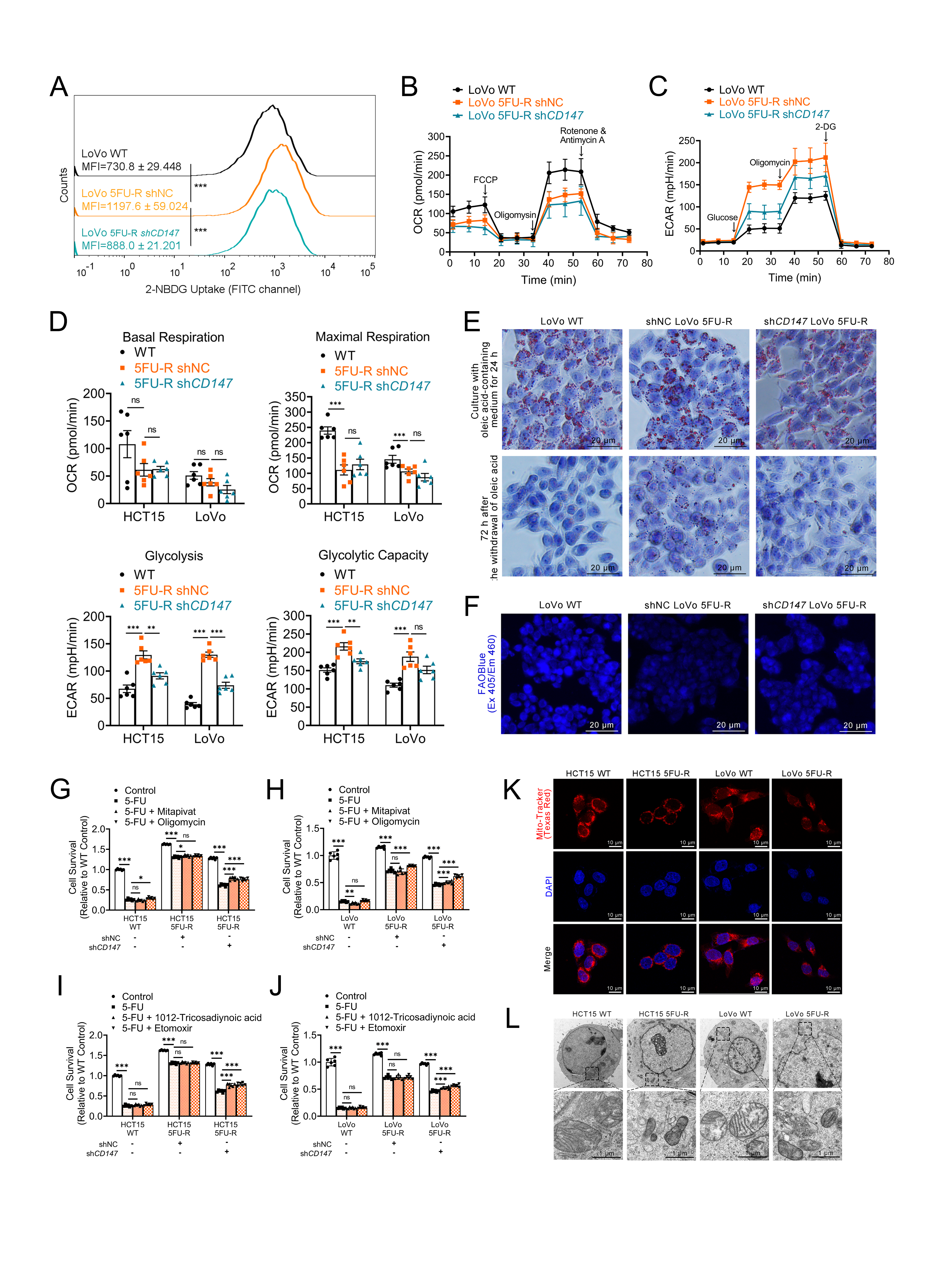
**

**Supplementary Figure S2 (related to Figure 2)**

1. 2-NBDG signals of WT and 5FU-R LoVo cells were quantified via flow cytometry. Results have been presented as mean MFI (FITC channel) ± SD.

(B, C) OCR and ECAR of WT and 5FU-R LoVo cells were measured using Seahorse XFe96. (B) FCCP (1 μM), Oligomycin (1.5 μM), and rotenone/antimycin A (0.5 μM) were successively added to measure OCR, and (C) glucose (100 mM), oligomycin (10 μM), and 2-DG (500 mM) were successively added to measure ECAR.

(D) Basal respiration, maximal respiration, glycolysis, and glycolytic capacity of 5FU-R cells compared to WT cells were measured using Seahorse XFe96.

(E) Oil Red O staining was performed to examine intracellular lipid droplets of LoVo cells. Scale bar = 20 μm.

(F) FAO was detected by using an FAOBlue probe and a fluorescence microscope. Scale bar = 20 μm.

(G, H) Indicated CRC cells were treated with 10^-5^ M 5-FU with 1 μM Mitapivat or 0.5 μM oligomycin for 48 h. Cell survival data were compared with those obtained for WT cells.

(I, J) Indicated CRC cells were treated with 10^-5^ M 5-FU with 0.2 μM 10,12-Tricosadiynoic acid or 1 μM Etomoxir for 48 h. Cell survival data were compared with those obtained for WT cells.

1. Mitochondrial contents were estimated by conducting Mito-Tracker staining. The mitochondria (red) and nucleus (blue); scale bar = 10 μm.

(L) Representative images of the ultrastructure of the mitochondria acquired via TEM. Scale bar = 1 μm.

Data are presented as mean ± SD. Data were compared by performing ANOVA (ns = not significant, * *p* < 0.05, ** *p* < 0.01, and *** *p* < 0.001). Source data are provided as a Source Data file.


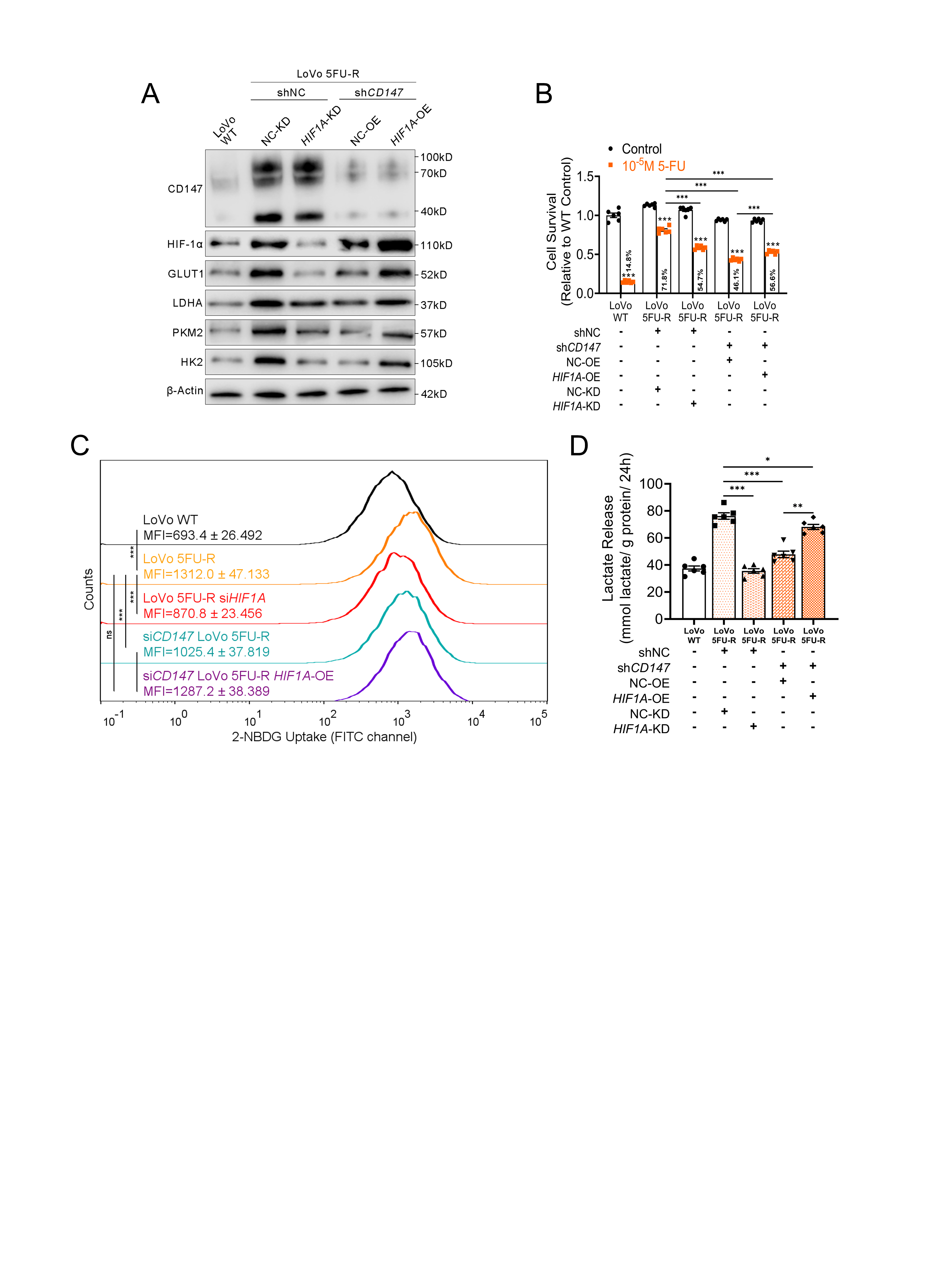
**Supplementary Figure S3 (related to Figure 3)**

1. Western blotting (WB) analyses of CD147, HIF-1α, GLUT1, and glycolytic enzymes in WT LoVo cells, 5FU-R LoVo cells subjected to treatment with sh*HIF1A* or control shRNA, and sh*CD147* LoVo 5FU-R cells subjected to treatment with *HIF1A*-OE lentivirus or control lentivirus.
2. Relative 5-FU sensitivity of the indicated LoVo cells, as determined via CCK-8 assays. Cell survival data were compared with those obtained for WT LoVo cells. The ratios of 5-FU treatment to control of each groups were calculated and have been indicated as percentages.
3. 2-NBDG signals were quantified via flow cytometry in WT LoVo cells, 5FU-R LoVo cells subjected to treatment with si*HIF1A* or control siRNA, and si*CD147* LoVo 5FU-R cells subjected to treatment with *HIF1A*-OE plasmids or control plasmids. Results have been presented as mean MFI (FITC channel) ± SD.
4. The cell culture supernatants of the indicated LoVo cells were assayed for lactate release using a colorimetric assay, and values have been normalized by cellular protein content.

Data are presented as mean ± SD. Data were compared by performing ANOVA (ns = not significant, * *p* < 0.05, ** *p* < 0.01, and *** *p* < 0.001). Source data are provided as a Source Data file.


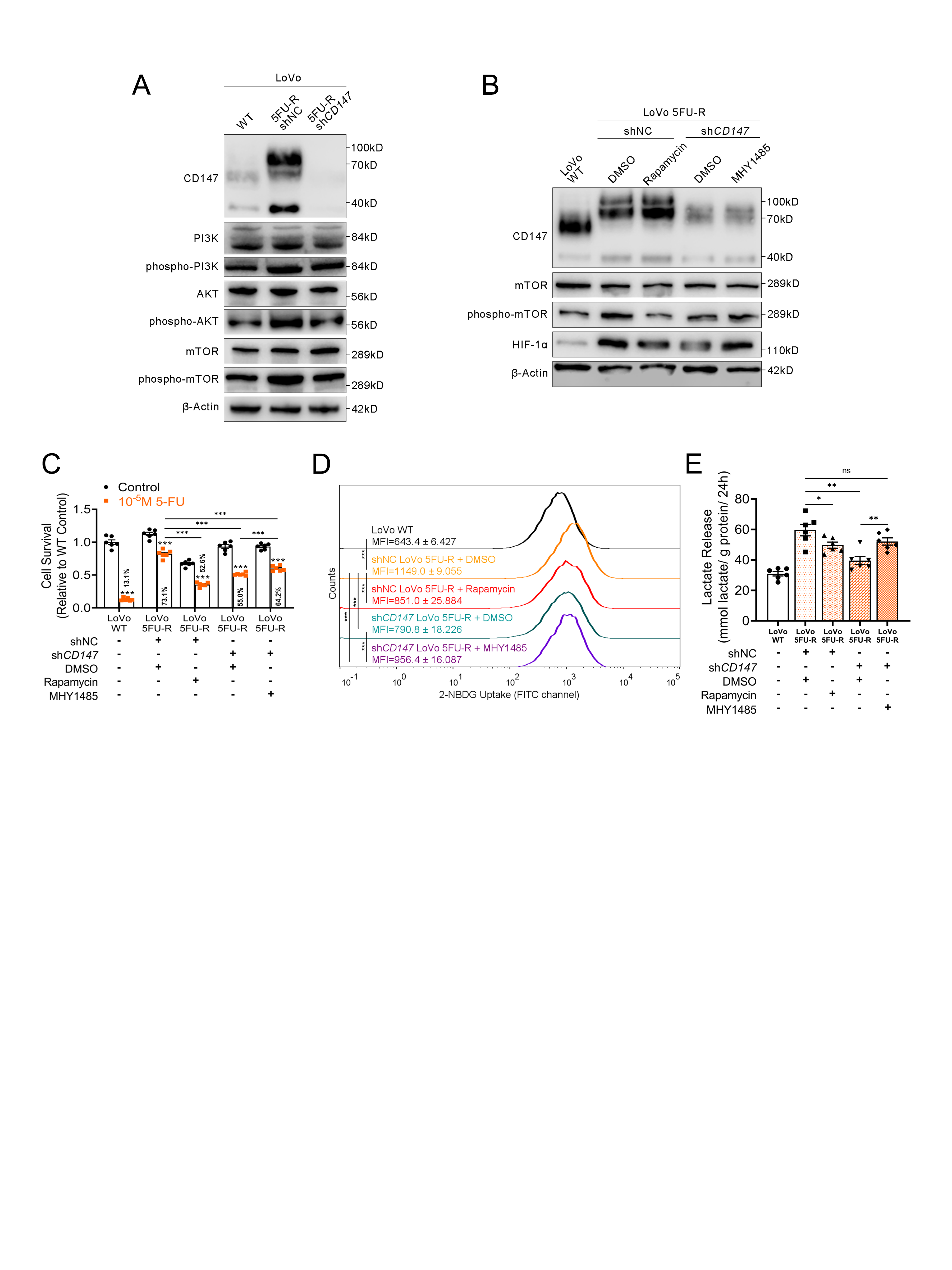
**Supplementary Figure S4 (related to Figure 4)**

1. Western blotting (WB) analyses of the expression levels of PI3K, phospho-PI3K, AKT, phospho-AKT, mTOR, and phospho-mTOR in WT, 5FU-R, and sh*CD147* 5FU-R LoVo cells.
2. WB analyses of CD147, mTOR, phospho-mTOR, and HIF-1α in WT LoVo cells, 5FU-R LoVo cells subjected to treatment with 50 nM rapamycin or DMSO (control), and sh*CD147* LoVo 5FU-R cells subjected to treatment with 10 μM MHY1485 or DMSO (control).
3. Relative 5-FU sensitivity of the indicated LoVo cells, as determined via CCK-8 assays. Cell survival data were compared with those obtained for WT LoVo cells. The ratios of 5-FU treatment to control of each groups were calculated and have been indicated as percentages.
4. 2-NBDG signals of the indicated LoVo cells were quantified via flow cytometry. Results have been presented as mean MFI (FITC channel) ± SD.
5. The cell culture supernatants of the indicated LoVo cells were assayed for lactate release using a colorimetric assay, and values have been normalized by cellular protein content.

Data are presented as mean ± SD. Data were compared by performing ANOVA (ns = not significant, * *p* < 0.05, ** *p* < 0.01, and *** *p* < 0.001). Source data are provided as a Source Data file.


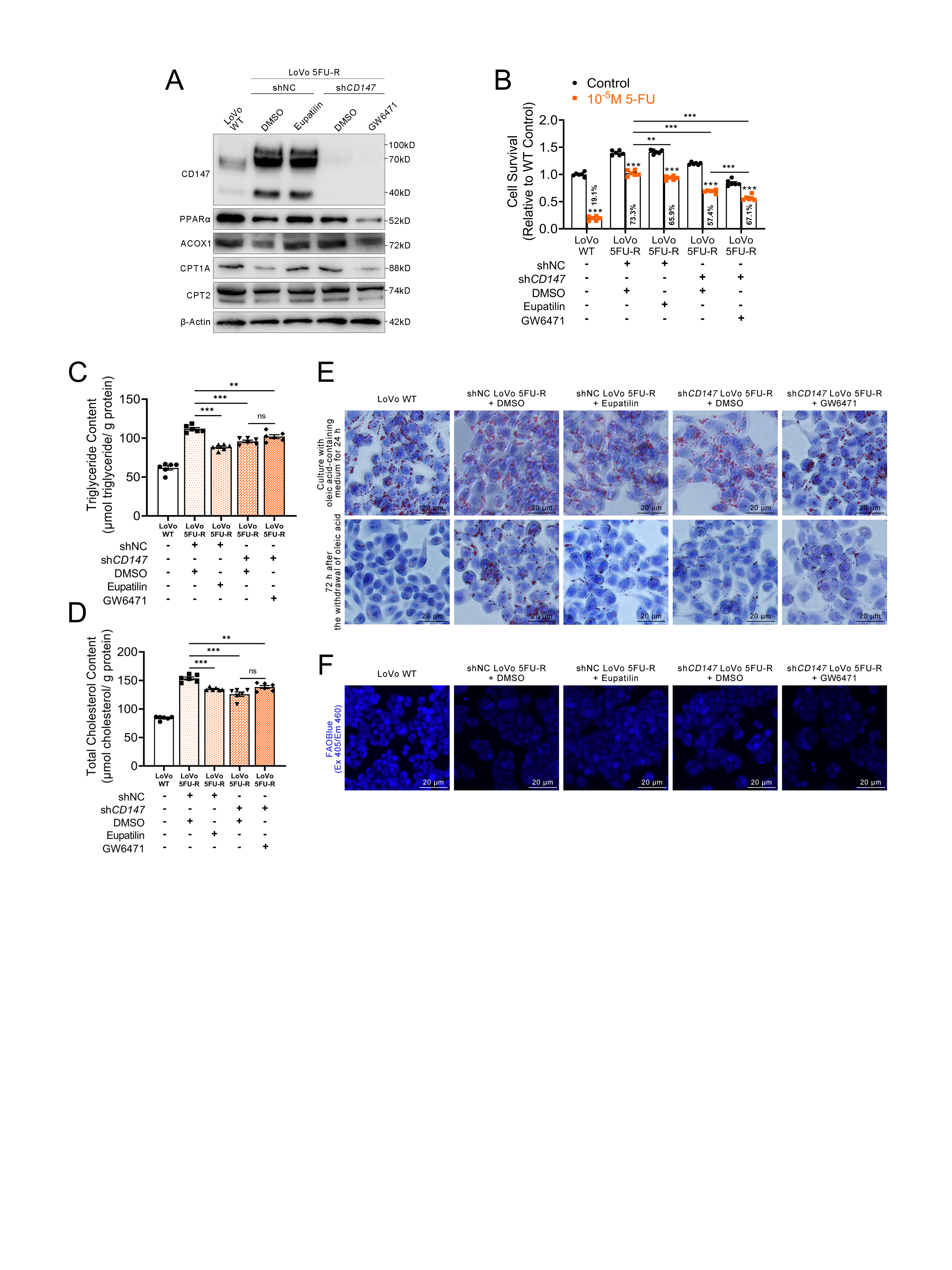
**Supplementary Figure S5 (related to Figure 5)**

1. Western blotting (WB) analyses of CD147, PPARα, and FAO-related enzymes in WT LoVo cells, 5FU-R LoVo cells subjected to treatment with 50 μM eupatilin or DMSO (control), and sh*CD147* LoVo 5FU-R cells subjected to treatment with 25 μM GW6471 or DMSO (control).
2. Relative 5-FU sensitivity of the indicated LoVo cells, as determined via CCK-8 assays. Cell survival data were compared with those obtained for WT LoVo cells. The ratios of 5-FU treatment to control of each groups were calculated and have been indicated as percentages.

(C, D) The cellular contents of (C) triglyceride and (D) total cholesterol of the indicated LoVo cells were assayed using a colorimetric assay, and values have been normalized by cellular protein content.

(E) Oil Red O staining was performed to examine intracellular lipid droplets of the indicated LoVo cells. Scale bar = 20 μm.

(F) FAO was detected by using an FAOBlue probe and a fluorescence microscope. Scale bar = 20 μm.

Data are presented as mean ± SD. Data were compared by performing ANOVA (ns = not significant, * *p* < 0.05, ** *p* < 0.01, and *** *p* < 0.001). Source data are provided as a Source Data file.


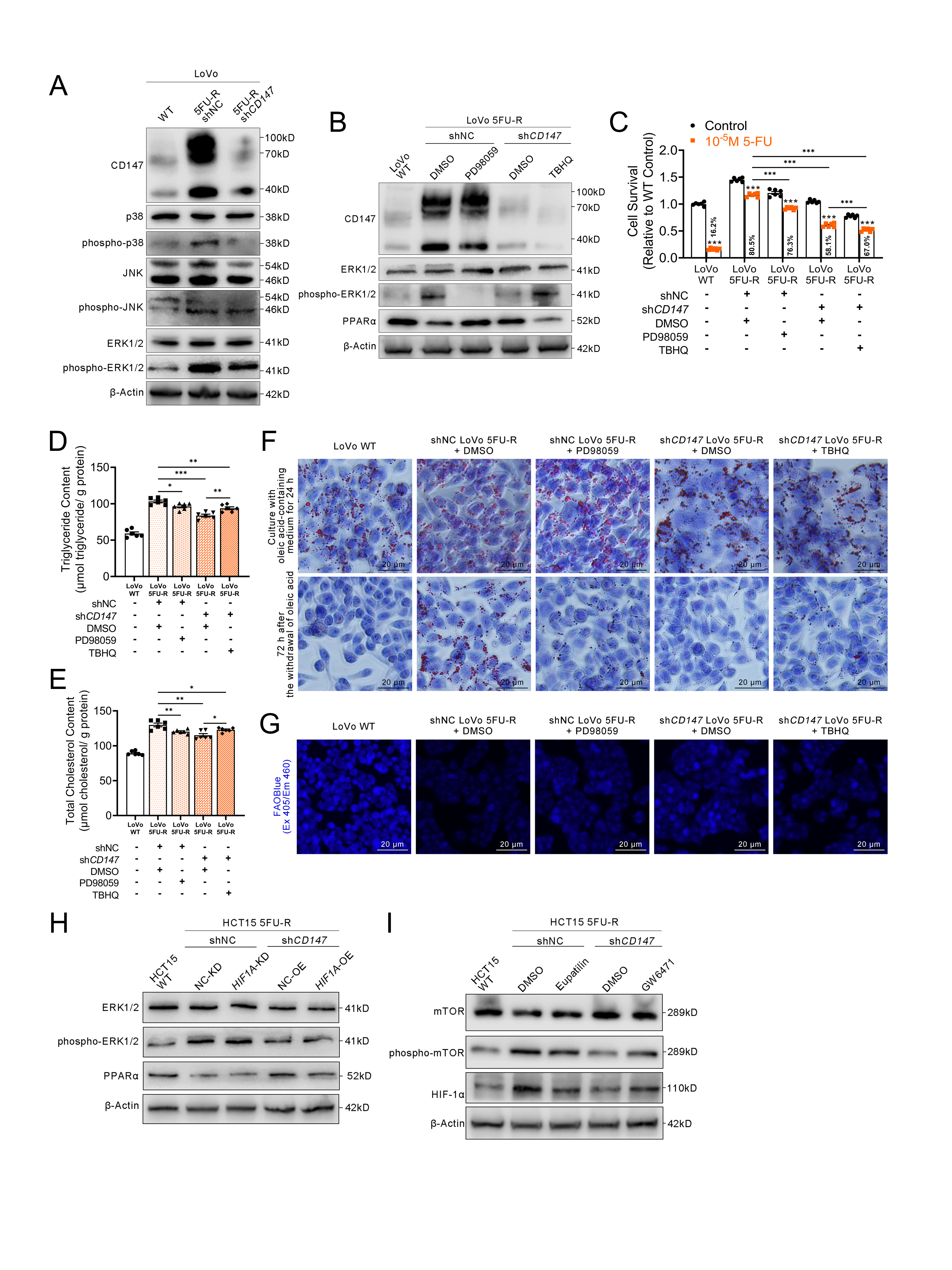


**Supplementary Figure S6 (related to Figure 6)**

1. Western blotting (WB) analyses of the expression levels of p38, phospho-p38, JNK, phospho-JNK, ERK, and phospho-ERK in WT, 5FU-R, and sh*CD147* 5FU-R LoVo cells.
2. WB analyses of CD147, ERK, phospho-ERK, and PPARα in WT LoVo cells, 5FU-R LoVo cells subjected to treatment with 50 μM PD98059 or DMSO (control), and sh*CD147* LoVo 5FU-R cells subjected to treatment with 20 μM TBHQ or DMSO (control).
3. Relative 5-FU sensitivity of the indicated LoVo cells, as determined via CCK-8 assays. Cell survival data were compared with those obtained for WT LoVo cells. The ratios of 5-FU treatment to control of each groups were calculated and have been indicated as percentages.

(D, E) The cellular contents of (D) triglyceride and (E) total cholesterol of the indicated LoVo cells were assayed using a colorimetric assay, and values have been normalized by cellular protein content.

(F) Oil Red O staining was performed to examine intracellular lipid droplets of the indicated LoVo cells. Scale bar = 20 μm.

(G) FAO was detected by using an FAOBlue probe and a fluorescence microscope. Scale bar = 20 μm.

(H) WB analyses of ERK1/2, phospho-ERK1/2 and PPARα in WT HCT15 cells, 5FU-R HCT15 cells subjected to treatment with sh*HIF1A* or control shRNA, and sh*CD147* HCT15 5FU-R cells subjected to treatment with *HIF1A*-OE lentivirus or control lentivirus.

(I) WB analyses of mTOR, phospho-mTOR and HIF-1α in WT HCT15 cells, 5FU-R HCT15 cells subjected to treatment with 50 μM eupatilin or DMSO (control), and sh*CD147* HCT15 5FU-R cells subjected to treatment with 25 μM GW6471 or DMSO (control).

Data are presented as mean ± SD. Data were compared by performing ANOVA (ns = not significant, * *p* < 0.05, ** *p* < 0.01, and *** *p* < 0.001). Source data are provided as a Source Data file.


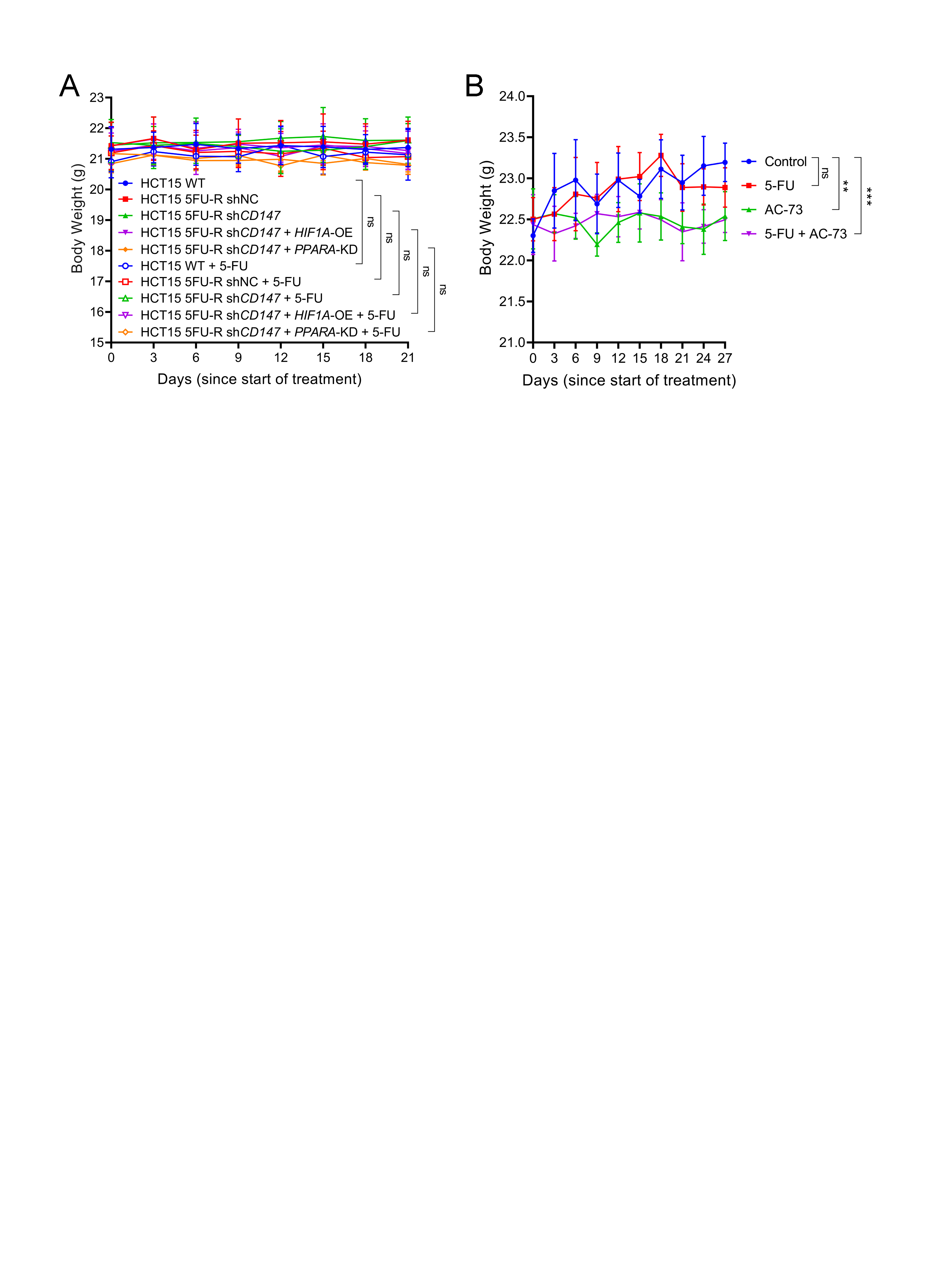
**Supplementary Figure S7 (related to Figure 7)**

1. Body weights of the indicated HCT15 cell-harboring mice, followed by treatment with 5-FU (administered intraperitoneally, 25 mg/kg, three times a week) or saline (control).
2. Body weights of PDX NOD/scid mice, followed by treatment with 5-FU (administered intraperitoneally, 25 mg/kg, three times a week), AC-73 (administered intraperitoneally, 25 mg/kg, daily), or saline (control).

Data are presented as mean ± SD. Data were compared by performing ANOVA (ns = not significant, * *p* < 0.05, ** *p* < 0.01, and *** *p* < 0.001). Source data are provided as a Source Data file.
