# Supplementary material for: CD147 Mediates 5-Fluorouracil Resistance in Colorectal Cancer by Reprogramming Glycolipid Metabolism
Source: Front Oncol. 2022 Jul 11;12:813852. doi: 10.3389/fonc.2022.813852 (PMC9309564; doi:10.3389/fonc.2022.813852)
Supplement: Supplementary file 2 [file DataSheet_2.docx]

**Supplementary Tables**

**Title:** CD147 mediates 5-fluorouracil resistance in colorectal cancer by reprogramming glycolipid metabolism

**Authors’ names:** ShuoHui Dong^1^, SongHan Li^1^, XiaoYan Wang^2^, Shuo Liang^3^, WenJie Zhang^1^, LinChuan Li^4^, Qian Xv^1^, BoWen Shi^1^, ZhiQiang Cheng^5^, Xiang Zhang^5^, MingWei Zhong^4^, GuangYong Zhang^4^, SanYuan Hu^1*^

**Authors’ affiliations:**

^1^Department of General Surgery, Shandong Qianfoshan Hospital, Cheeloo College of Medicine, Shandong University, Jinan, China.

^2^Department of Neonatology, Weifang Yidu Central Hospital, Weifang, China.

^3^Department of Otolaryngology-Head and Neck Surgery, Shandong Provincial ENT Hospital, Cheeloo College of Medicine, Shandong University, Jinan, China.

^4^Department of General Surgery, The First Affiliated Hospital of Shandong First Medical University, Jinan, China

^5^Department of General Surgery, Qilu Hospital, Cheeloo College of Medicine, Shandong University, Jinan, China.

**Corresponding author:** SanYuan Hu, Department of General Surgery, Shandong Qianfoshan Hospital, Cheeloo College of Medicine, Shandong University, Jinan, Shandong 250014, China. Email: drsanyuanhu@163.com. Tel.: +86 18663738139.

**Supplementary Table 1. Reagent used in this study.**

| **Reagent** | **Source** | **Identifier** |
| --- | --- | --- |
| anti-CD147 antibody | Abcam | Cat# ab108308 |
| anti-HIF-1α antibody | Abcam | Cat# ab179483 |
| anti-GLUT1 antibody | Abcam | Cat# ab40084 |
| anti-LDHA antibody | Proteintech | Cat# 66287-1-Ig |
| anti-HK2 antibody | Abcam | Cat# ab209847 |
| anti-PKM2 antibody | CST | Cat# 4053 |
| anti-PI3K antibody | Affinity | Cat# AF6241 |
| anti-phospho-PI3K antibody | Affinity | Cat# AF3241 |
| anti-AKT antibody | Abcam | Cat# ab179463 |
| anti-phospho-AKT antibody | Abcam | Cat# ab38449 |
| anti-mTOR antibody | HUABIO | Cat# ET1608-5 |
| anti-phospho-mTOR antibody | Abcam | Cat# ab109268 |
| anti-PPARα antibody | Abcam | Cat# ab245119 |
| anti-ACOX1 antibody | Proteintech | Cat# 10957-1-AP |
| anti-CPT1A antibody | Proteintech | Cat# 15184-1-AP |
| anti-CPT2 antibody | Proteintech | Cat# 26555-1-AP |
| anti-p38 antibody | HUABIO | Cat# ET1602-26 |
| anti-phospho-p38 antibody | HUABIO | Cat# ER2001-52 |
| anti-JNK antibody | HUABIO | Cat# ET1601-28 |
| anti-phospho-JNK antibody | CST | Cat# 4668 |
| anti-ERK1/2 antibody | HUABIO | Cat# ET1601-29 |
| anti-phospho-ERK1/2 antibody | CST | Cat# 4370 |
| anti-Ki-67 antibody | Proteintech | Cat# 27309-1-AP |
| anti-β-Actin antibody | Proteintech | Cat# 66009-1-Ig |
| HRP-conjugated Affinipure Goat Anti-Rabbit IgG(H+L) | Proteintech | Cat# SA00001-2 |
| HRP-conjugated Affinipure Goat Anti-Mouse IgG(H+L) | Proteintech | Cat# SA00001-1 |
| CoraLite594 – conjugated Goat Anti-Rabbit IgG(H+L) | Proteintech | Cat# SA00013-4 |
| HRP-conjugated anti-rabbit/mouse IgG | ZSGB-BIO | Cat# PV-9000 |

**Supplementary Table 2. Reagents used in this study.**

| **Reagent** | **Source** | **Identifier** |
| --- | --- | --- |
| 5-Fluorouracil (5-FU) | Abcam | Cat# ab142387 |
| 2-NBDG | MedChemExpress | Cat# HY-116215 |
| FAOBlue (Fatty acid oxidation detection reagent) | FUJIFILM | Cat# FDV-0033 |
| TRIzol reagent | TaKaRa | Cat# 279506 |
| RIPA lysis buffer（Strong） | CWBIO | Cat# CW2333 |
| 100 × Protease inhibitor cocktail | CWBIO | Cat# CW2200 |
| 100 × Phosphatase inhibitor cocktail | CWBIO | Cat# CW2383 |
| 5 × Loading buffer | CWBIO | Cat# CW0027 |
| PageRuler prestained protein ladder | Thermo Scientific | Cat# 26616 |
| Lipofectamine RNA iMAX reagent | Life technologies | Cat# 13778030 |
| Lipofectamine 3000 | Life technologies | Cat# L3000015 |
| Rapamycin (a mTOR inhibitor) | MedChemExpress | Cat# HY-10219 |
| MHY1485 (a mTOR activator) | MedChemExpress | Cat# HY-B0795 |
| Eupatilin (a PPARα agonist) | MedChemExpress | Cat# HY-N0783 |
| GW6471 (a PPARα antagonist) | MedChemExpress | Cat# HY-15372 |
| PD98059 (an ERK1/2 signaling inhibitor) | MedChemExpress | Cat# HY-12028 |
| TBHQ (an ERK activator) | MedChemExpress | Cat# HY-100489 |
| AC-73 (a CD147 inhibitor) | MedChemExpress | Cat# HY-122214 |
| Mitapivat (a selective PKM2 activator) | Selleck | Cat# S6508 |
| Oligomycin (an ATP synthase inhibitor) | MedChemExpress | Cat# HY-N6782 |
| 10,12-Tricosadiynoic acid (an ACOX1 inhibitor) | MedChemExpress | Cat# HY-135425 |
| Etomoxir sodium salt (a CPT-1 inhibitor) | Selleck | Cat# S8244 |

**Supplementary Table 3. Critical commercial assays used in this study.**

| **Reagent** | **Source** | **Identifier** |
| --- | --- | --- |
| Cell counting kit-8 | MedChemExpress | Cat# HY-K0301 |
| 10% TGX Stain-Free™ FastCast™ Acrylamide kit | Bio-rad | Cat# 1610183 |
| Chemiluminescent HRP substrate kit | Millipore | Cat# WBKLS0100 |
| ReverTra Ace qPCR RT Kit | TOYOBO | Cat# FSQ-101 |
| SYBR Green Realtime PCR Master Mix | TOYOBO | Cat# QPK-201 |
| Mitochondrial staining kit | Abcam | Cat# ab112145 |
| Lactate release assay kit | KeyGEN BioTECH | Cat# KGT023 |
| Seahorse XF cell mito stress test kit | Agilent | Cat# 103010-100 |
| Seahorse XF glycolysis stress test kit | Agilent | Cat# 103020-100 |
| Triglyceride assay kit | Solarbio | Cat# BC0620 |
| Total cholesterol assay kit | Solarbio | Cat# BC1980 |
| Oil Red O stain kit (for cultured cells) | Solarbio | Cat# G1262 |

**Supplementary Table 4. Sequences targeting genes.**

| **Construction of lentivirus vectors** | | |
| --- | --- | --- |
| **Gene** | **Target sequence** | **Vector** |
| sh*CD147* | CATCATACACTTCCTTCTT | hU6-MCS-Ubiquitin-EGFP-IRES-puromycin (GV248, Jikai Gene, China) |
| sh*HIF1A* | aaTGTGAGTTCGCATCTTGAT | hU6-MCS-Ubiquitin-EGFP-IRES-puromycin (GV248, Jikai Gene, China) |
| sh*PPARA* | agTGGAGCATTGAACATCGAA | hU6-MCS-CBh-gcGFP-IRES-puromycin (GV493, Jikai Gene, China) |
| Control 1 | Cat# CON077, Negative Control to sh*CD147* and sh*HIF1A* | hU6-MCS-Ubiquitin-EGFP-IRES-puromycin (GV248, Jikai Gene, China) |
| Control 2 | Cat# CON313, Negative Control to sh*PPARA* | hU6-MCS-CBh-gcGFP-IRES-puromycin (GV493, Jikai Gene, China) |
| Control 3 | Cat# CON335, Negative Control to *HIF1A*-OE | Ubi-MCS-3FLAG-CBh-gcGFP-IRES-puromycin (GV492, Jikai Gene, China) |
| **siRNA sequences targeting genes** | | |
| si*CD147* | 5’-GUUCUUCGUGAGUUCCUCtt-3’, 3’-dTdTCAAGAAGCACUCAAGGAG-5’ | |
| si*HIF1A* | 5’-UACUCAGAGCUUUGGAUCAAGUUAAtt-3’, 3’-ttAUGAGUCUCGAAACCUAGUUCAAUU-5’ | |

**Supplementary Table 5. Clinicopathological data.**

| **Patient Characteristics** | **Category** | **Frequency (number)** |
| --- | --- | --- |
| ***Cohort 1***  no chemotherapy group (without preoperative chemotherapy) | | |
| Age (years) | < 40 | 0.00% (0) |
|  | 40-49 | 26.67% (4) |
|  | 50-59 | 20.00% (3) |
|  | 60-69 | 33.33% (5) |
|  | 70-79 | 20.00% (3) |
|  | >80 | 0.00% (0) |
| Gender | Male | 53.33% (8) |
|  | Female | 46.67% (7) |
| T stage | T1 | 0.00% (0) |
|  | T2 | 6.67% (1) |
|  | T3 | 60.00% (9) |
|  | T4a | 33.33% (5) |
|  | T4b | 0.00% (0) |
| N stage | N0 | 0.00% (0) |
|  | N1a | 20.00% (3) |
|  | N1b | 13.33% (2) |
|  | N1c | 20.00% (3) |
|  | N2a | 26.67% (4) |
|  | N2b | 20.00% (3) |
| M stage | M0 | 93.33% (14) |
|  | M1 | 6.67% (1) |
| TNM stage | III | 93.33% (14) |
|  | IV | 6.67% (1) |
| ***Cohort 2***  response group (good response to preoperative fluorouracil analog–based chemotherapy) | | |
| Age (years) | < 40 | 0.00% (0) |
|  | 40-49 | 13.33% (2) |
|  | 50-59 | 33.33% (5) |
|  | 60-69 | 53.33% (8) |
|  | 70-79 | 0.00% (0) |
|  | >80 | 0.00% (0) |
| Gender | Male | 66.67% (10) |
|  | Female | 33.33% (5) |
| T stage | T1 | 6.67% (1) |
|  | T2 | 0.00% (0) |
|  | T3 | 60.00% (9) |
|  | T4a | 33.33% (5) |
|  | T4b | 0.00% (0) |
| N stage | N0 | 13.33% (2) |
|  | N1a | 13.33% (2) |
|  | N1b | 6.67% (1) |
|  | N1c | 13.33% (2) |
|  | N2a | 33.33% (5) |
|  | N2b | 20.00% (3) |
| M stage | M0 | 80.00% (12) |
|  | M1 | 20.00% (3) |
| TNM stage | III | 80.00% (12) |
|  | IV | 20.00% (3) |
| Primary chemotherapy regimens | 5-FU | 13.33% (2) |
|  | Capecitabine | 20.00% (3) |
|  | FOLFOX | 40.00% (6) |
|  | CAPEOX | 6.67% (1) |
|  | FOLFIRI | 13.33% (2) |
|  | FOLFOXIRI | 6.67% (1) |
|  | XELOX | 0.00% (0) |
| ***Cohort 3***  no response group (poor response to preoperative fluorouracil analog–based chemotherapy) | | |
| Age (years) | < 40 | 8.33% (1) |
|  | 40-49 | 25.00% (3) |
|  | 50-59 | 33.33% (4) |
|  | 60-69 | 16.67% (2) |
|  | 70-79 | 8.33% (1) |
|  | >80 | 8.33% (1) |
| Gender | Male | 58.33% (7) |
|  | Female | 41.67% (5) |
| T stage | T1 | 0.00% (0) |
|  | T2 | 0.00% (0) |
|  | T3 | 33.33% (4) |
|  | T4a | 66.67% (8) |
|  | T4b | 0.00% (0) |
| N stage | N0 | 0.00% (0) |
|  | N1a | 8.33% (1) |
|  | N1b | 50.00% (6) |
|  | N1c | 8.33% (1) |
|  | N2a | 8.33% (1) |
|  | N2b | 25.00% (3) |
| M stage | M0 | 75.00% (9) |
|  | M1 | 25.00% (3) |
| TNM stage | III | 75.00% (9) |
|  | IV | 25.00% (3) |
| Primary chemotherapy regimens | 5-FU | 8.33% (1) |
|  | Capecitabine | 8.33% (1) |
|  | FOLFOX | 41.67% (5) |
|  | CAPEOX | 0.00% (0) |
|  | FOLFIRI | 33.33% (4) |
|  | FOLFOXIRI | 0.00% (0) |
|  | XELOX | 8.33% (1) |

**Supplementary Table 6. Primers used in RT-qPCR.**

| **Gene** | **Forward Sequence** | **Reverse Sequence** |
| --- | --- | --- |
| *CD147* | GGCTGTGAAGTCGTCAGAACAC | ACCTGCTCTCGGAGCCGTTCA |
| *HIF1A* | TATGAGCCAGAAGAACTTTTAGGC | CACCTCTTTTGGCAAGCATCCTG |
| *PPARA* | TCGGCGAGGATAGTTCTGGAAG | GACCACAGGATAAGTCACCGAG |
| *ACTB* | CACCATTGGCAATGAGCGGTTC | AGGTCTTTGCGGATGTCCACGT |
